# Intranasal Vaccine Adjuvants and Delivery Platforms: From Barrier Mechanisms to Clinical Translation

**DOI:** 10.3390/vaccines14040295

**Published:** 2026-03-26

**Authors:** Shunyu Yao, Zhe Zhai, Liqi Liao, Linglin Zhong, Chenyu Shi, Yong-Xian Cheng, Xuhan Liu

**Affiliations:** 1 School of Pharmacy, Guangdong Pharmaceutical University, Guangzhou 510006, China; 2 Guangdong Provincial Key Laboratory of Chinese Medicine Ingredients and Gut Microbiomics, Institute for Inheritance-Based Innovation of Chinese Medicine, Marshall Laboratory of Biomedical Engineering, School of Pharmacy, Shenzhen University Medical School, Shenzhen University, Shenzhen 518055, China; 3 College of Basic Medicine, Yunnan University of Chinese Medicine, Kunming 650500, China

**Keywords:** intranasal vaccine, mucosal immunity, adjuvant, vaccine carrier, clinical translation

## Abstract

As a non-invasive mucosal immunization strategy, intranasal vaccines are highly promising for preventing respiratory infectious diseases. Among them, recombinant subunit vaccines represent a safe and ideal option, as they induce targeted mucosal immunity without the safety risks associated with live-vectored or nucleic acid vaccines. However, nasal mucosal defenses rapidly clear antigens before immune activation, limiting protective efficacy. Therefore, intranasal vaccine adjuvants—key regulators of immune response intensity, duration, and type—are essential to overcome mucosal tolerance and improve immunogenicity. Based on a systematic search and analysis of 127 peer-reviewed articles (2010–2026) in PubMed, Web of Science, and Embase, this study comprehensively summarizes the mechanisms, applications, and limitations of existing and candidate adjuvants for intranasal vaccines. This review systematically categorizes and discusses the nasal mucosal barrier, major adjuvant types (e.g., pattern recognition receptor agonists, cytokine adjuvants, and carrier adjuvants), and their mechanisms of action. It also identifies key bottlenecks: insufficient mucosal targeting, inconsistent global safety evaluation standards for adjuvants, and interference from pre-existing antibodies in humans. Furthermore, this review highlights future development directions, including biomimetic adjuvants, pH-responsive nanoadjuvants, and thermostable vaccine formulations. This systematic review clarifies key scientific and technical barriers in intranasal vaccine adjuvant development. The findings provide valuable references for advancing the translation of intranasal vaccines from emergency countermeasures to routine, accessible preventive tools for respiratory infectious diseases.

## 1. Introduction

The development of respiratory mucosal vaccines is facing unprecedented opportunities and challenges. In the global public health landscape, respiratory infections caused by pathogenic microorganisms pose a significant threat: according to data from the World Health Organization (WHO), such infections account for approximately 6% of the global disease burden, resulting in around 17 million deaths annually. Among these, children under 5 years of age are particularly susceptible, with 6.6 million deaths each year attributed to respiratory viral infections. From the 1918 H1N1 influenza pandemic to the recent pandemic caused by severe acute respiratory syndrome coronavirus 2 (SARS-CoV-2), the continuous evolution, emergence, and re-emergence of respiratory viruses have underscored the urgency of developing novel preventive strategies, and intranasal immunization has become a key direction owing to its unique advantages [1,2,3].

**Figure 1:**
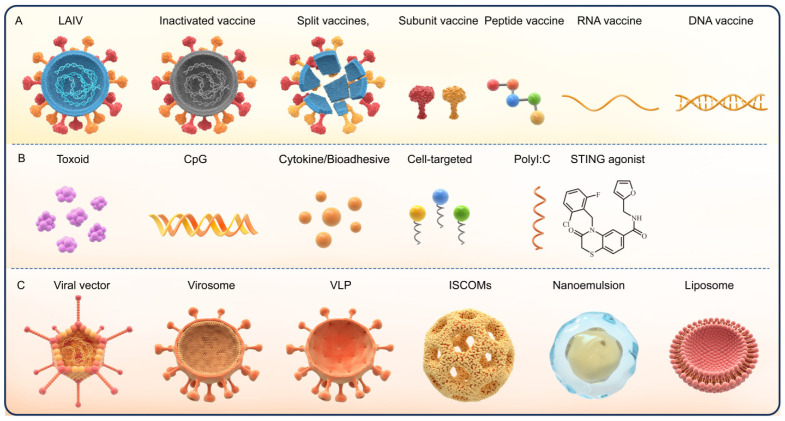
Classification of intranasal vaccine components and adjuvants [reprinted from reference [16], which is licensed under CC BY 4.0]. (**A**) Vaccine modalities, categorized by antigen form and preparation process, include live attenuated influenza vaccine (LAIV), inactivated vaccine, split vaccines, subunit vaccine, peptide vaccine, RNA vaccine, and DNA vaccine. (**B**). Adjuvant types, classified by functional mechanism and molecular target, encompass toxoid, CpG oligodeoxynucleotide (CpG), cytokine/bioadhesive agents, cell-targeted adjuvants, polyinosinic:polycytidylic acid (Poly I:C), and stimulator of interferon genes (STING) agonists. (**C**). Delivery systems and particulate carriers for intranasal vaccines consist of viral vector, virosome, virus-like particle (VLP), immune-stimulating complexes (ISCOMs), nanoemulsion, and liposome, which enhance stability, mucosal uptake, and immune activation while mitigating mucociliary clearance (MCC) challenges.

As a vaccine delivery method, intranasal immunization not only overcomes the limitations of traditional injectable vaccines but also establishes a dual immune defense line at the portal of infection by inducing local mucosal and systemic responses to vaccines, specifically by stimulating the production of secretory immunoglobulin A (sIgA), thus constructing the first line of protective barrier for the body. As the core effector molecule of mucosal immunity, sIgA lacks the ability to directly kill pathogens, but it can effectively block pathogen invasion through neutralization and agglutination, as well as promote their elimination from the body via the mucociliary clearance (MCC) system. This mechanism holds special significance for preventing a variety of mucosa-infecting pathogens [4]. Many pathogens, including human immunodeficiency virus (HIV), SARS-CoV-2, influenza virus, rotavirus, and Vibrio cholerae, infect hosts through mucosal surfaces; hence, sIgA plays an irreplaceable role in mucosal immunity [5]. Meanwhile, as the core component regulating immune responses, adjuvants are precisely the key to enhancing intranasal immunization efficacy and inducing robust sIgA responses. Intranasal vaccines consist of two core components: vaccine modalities with different antigen forms and adjuvants that regulate immune responses. The mainstream types of both components are systematically classified in Figure 1. Vaccine modalities range from traditional live attenuated and inactivated vaccines to novel genetic engineering vaccines (e.g., RNA, DNA, and recombinant vector vaccines) and recombinant subunit vaccines—the latter has become the research focus of intranasal vaccine development due to its high safety, clear antigen composition, and low risk of pathogenic reversion or nucleic acid integration. However, intranasal subunit vaccines face inherent bottlenecks in clinical application: their poor ability to cross the nasal mucosal physiological barrier leads to rapid antigen degradation and clearance, and the lack of intrinsic immunogenicity results in weak mucosal immune responses, making exogenous adjuvants and efficient delivery systems essential prerequisites for their clinical translation. Adjuvants are divided into immunostimulatory adjuvants (e.g., TLR agonists and STING agonists), bioadhesive adjuvants, and cell-targeted adjuvants according to their functional mechanisms. This classification provides a clear framework for the subsequent analysis of adjuvant action mechanisms and delivery system optimization (Figure 1).

However, the challenge of delivering vaccine components across the mucosal barrier has long hindered the development of mucosal vaccines, and the essence of this barrier is related to the physiological environment of the mucosal segment. The proteolytic enzyme degradation and acidic environments on the mucosal surface may lead to rapid antigen loss, along with a high MCC rate and multiple physical and biological barriers at airway mucosal sites [6,7]. These factors not only impede vaccine uptake but also impose stringent requirements on adjuvants—they should ideally possess core characteristics of resisting mucosal clearance, efficiently penetrating barriers, and specifically activating mucosal immune responses. Currently, the number of approved mucosal vaccines is extremely limited: only 15 mucosal vaccines have been licensed for human use worldwide, among which four are administered via the intranasal route and one via aerosolization of liquid vaccines using a nebulizer (products include FluMist/Fluenz [8,9]; Nasovac-S [10,11]; pandemic influenza vaccine H5N1 by AstraZeneca [12]; and iNCOVACC (BBV154) [13]; another one is administered by converting a liquid vaccine into an aerosol with a nebulizer (Convidecia Air (Ad5-nCoV-IH)) [14]. Notably, most of these approved vaccines are based on live attenuated viruses or inactivated whole-cell components [15]. Although some achieve mucosal immune effects, they carry potential safety risks such as possible virulence reversion of live attenuated viruses and incomplete inactivation of inactivated whole cells, which limit their application in vulnerable populations such as children and the elderly. In contrast, recombinant subunit vaccines, as a safe and controllable vaccine form, have become the ideal candidate for intranasal vaccination due to their non-infectiousness, clear antigen targeting, and good batch consistency. However, intranasal subunit vaccines face particularly prominent challenges of insufficient mucosal immunogenicity and poor barrier penetration ability due to their low molecular weight and weak mucosal adhesion—thus, developing highly effective, low-toxicity adjuvants and targeted delivery systems tailored for intranasal subunit vaccines is urgently needed to address this predicament and accelerate their clinical translation.

The physical barriers, biochemical barriers (enzymatic degradation), and immunological barriers of the nasal mucosa [16,17,18,19,20] not only pose core challenges to vaccine delivery but also serve as the fundamental basis for designing ideal intranasal vaccine adjuvants, which are expected to resist mucosal clearance of the ideal intranasal vaccine adjuvants by resisting mucosal clearance, efficiently penetrating barriers, and specifically activating mucosal immune responses. To systematically summarize the current research progress in addressing these challenges, particularly the innovative breakthroughs in the field of adjuvants, this review retrieved and compiled 127 relevant studies published between 2010 and 2026 from databases including PubMed, Web of Science, and CNKI. Prior to discussing adjuvant-related delivery strategies and mechanisms, Section 2 first systematically reviews the respiratory mucosal physiology (including barrier structure and immune cell function) to lay a theoretical foundation for subsequent adjuvant research. Centering on adjuvant optimization, this review will then comprehensively elaborate on the delivery strategies and mechanisms of action for nasal mucosal vaccines from three dimensions, namely innovation in adjuvant mechanisms of action, adjuvant–vector synergetic engineering, and upgrading of adjuvant delivery systems, with the aim of providing references for the development of efficient and safe intranasal vaccines.

## 2. Respiratory Mucosal Barrier: Structure and Function

The efficacy of intranasal vaccines is influenced by multiple factors and fundamentally regulated by the complex defense mechanisms of the respiratory mucosal barrier—which serves as the core target of vaccine action. The adhesion capacity of an adjuvant to the mucosa is closely correlated with its mucosal penetration efficiency: moderate and specific mucoadhesion can prolong the effective retention time of the adjuvant at the target site, creating favorable conditions for barrier penetration. However, excessive non-specific adhesion tends to induce local agglomeration of the adjuvant on the mucosal surface. This agglomeration not only hinders the effective contact between active components and the mucosal epithelial layer but also activates the respiratory mucociliary clearance (MCC) mechanism, accelerating the removal of aggregated materials and thereby leading to a relative reduction in actual mucosal penetration efficiency. Conversely, insufficient mucoadhesion may result in rapid clearance of the adjuvant before it can penetrate the barrier, also limiting its delivery efficacy. This relationship indicates that overcoming barrier challenges must be the primary step in vaccine design. The respiratory mucosal barrier is not a single structure but an integrated system composed of physical, chemical, and immunological components, all of which work synergistically to prevent pathogen invasion. This section will elaborate on the key components of this barrier: first, the MCC apparatus that rapidly removes foreign particulates; second, the epithelial tight junctions (TJs) and mucus layer that restrict substance penetration; and finally, the resident innate immune cells located beneath the epithelial layer. A thorough understanding of these barriers is paramount, as they represent the core challenges that effective intranasal vaccine adjuvants and delivery systems must overcome to induce protective immunity. Adjuvants capable of addressing these challenges need to possess core capabilities adapted to the barrier characteristics: they can extend the effective duration of action by optimizing the balance between moderate specific mucoadhesion and targeted mucosal retention (avoiding excessive non-specific adhesion-induced local agglomeration), enhance mucosal barrier penetration efficiency without disrupting the physiological homeostasis of the nasal mucosa, and precisely activate mucosal immune responses at the target site without triggering excessive local or systemic inflammatory reactions.

### 2.1. Ciliary Movement

MCC is the primary and crucial innate defense mechanism of the nasal cavity. Through the coordinated beating of cilia, it propels the mucus layer toward the pharyngeal end, a direction distinct from the ciliary movement in the lower respiratory tract that drives mucus toward the tracheal and bronchial distal ends, thereby entrapping and expelling inhaled particulates and pathogens and maintaining the nasal cavity in a relatively clean state. MCC also maintains the balance of the liquid layer on the nasal airway surface by regulating mucus secretion, hydration status, and ion transport to ensure the normal function of cilia. In cases of nasal infection or inflammation, the MCC mechanism can interact with the nasal mucosal immune system to participate in local immune regulation—mucus flow mediated by ciliary beating modulates the spatial distribution of immune cells (e.g., dendritic cells and mast cells) and the diffusion of immune mediators (e.g., cytokines and antimicrobial peptides) at the nasal mucosal surface, thereby fine-tuning the initiation and amplitude of innate and adaptive immune responses [17]. These multiple roles collectively protect the nasal cavity from harm and maintain a healthy nasal environment [17,18].

### 2.2. Tight Junctions (TJs) and Mucus Layer

If the coordinated beating of cilia is the core of the nasal cavity’s dynamic physical clearance capacity, then the structural integrity of the respiratory epithelial barrier—particularly the TJs and the overlying mucus layer—forms a static yet equally critical line of defense against pathogen invasion.

#### 2.2.1. Respiratory Epithelial Cells and Tight Junctions

Airway epithelial cells mainly consist of three core types: basal cells (assisting in anchoring epithelial cells to the underlying matrix), ciliated cells (clearing mucosal secretions and foreign substances from the lungs to the larynx directionally), and secretory cells (including goblet, serous, club, and neuroendocrine cells that secrete mucins to bind microorganisms, inflammatory cells, or pollutant particles for airway cleansing). Functionally, they not only perform physical foreign body clearance and airway cleaning but also act as a key immune barrier [19]. When stimulated by external allergens, their surface pattern recognition receptors (PRRs) are activated, triggering signal transduction that promotes the transcription of NF-κB and other transcription factors, thereby producing interferons, pro-inflammatory cytokines, and epithelial-derived cytokines to induce Th2-type inflammatory responses. This immune barrier function is closely linked to their special structure: airway epithelial cells line the airway surface, with junctions (mainly TJs, adherens junctions, and desmosomes) maintaining barrier integrity and separating subepithelial tissues from external stimuli [20]. Among these, tight junctions (TJs) are the most apical cell junctions and are composed of transmembrane proteins including claudins, the TAM family (tricellulin, MarvelD2, and angulin)—a group of transmembrane proteins specifically localized at tricellular tight junctions (tTJs) that mediate barrier function at the intersection of three epithelial cells—and occludin [21], as well as cytoplasmic scaffolding proteins (ZO-1, -2, and -3), and they exhibit selective permeability to macromolecules and ions. As a critical component of epithelial junctions, TJs are vital for maintaining epithelial integrity, determining intercellular permeability, performing immune surveillance to prevent foreign particle invasion, and participating in cell proliferation and differentiation mechanisms [22,23]. To target the TJ barrier, ideal adjuvants should possess the ability to gently regulate cell junctions: by specifically targeting the regulatory pathways of tight junction-related proteins, they can transiently and reversibly increase intercellular permeability to create conditions for vaccine component penetration; meanwhile, it is necessary to rationally control the intensity of regulation to avoid irreversible damage to the epithelial barrier or abnormal inflammatory responses, thus ensuring that the normal physiological functions of mucosal tissues remain unaffected.

#### 2.2.2. Respiratory Mucus Layer

If TJs serve as the “sealing gate” that fortifies the structural integrity of the airway epithelial barrier, the overlying respiratory mucus layer acts as a “protective blanket” that provides both physical entrapment and immune defense against invading pathogens.

In normal individuals, respiratory mucus is a viscoelastic hydrogel primarily composed of 95–98% water, with the remaining solid fraction (2–5%) consisting of high-molecular-weight mucin polymers (predominantly MUC5AC and MUC5B), electrolytes (salt), globular proteins, antimicrobial peptides, and lipids [24]. As a physical barrier, the respiratory mucus layer can adhere to and trap inhaled dust, pollen, and smoke particles, as well as pathogens such as bacteria and viruses, preventing them from directly contacting airway epithelial cells and thus playing a protective role. It can also recognize and kill pathogens through components like immunoglobulins, antimicrobial peptides, and proteins to initiate immune responses and resist pathogen invasion. Additionally, the mucus layer provides a suitable environment for ciliary beating. Relying on the MCC system, ciliary beating propels mucus toward the pharynx, transporting trapped foreign bodies and pathogens to the pharynx, which are then expelled from the body through coughing or swallowing. Furthermore, the water content in mucus can regulate airway humidity, keep the airway surface moist, maintain the integrity and normal function of airway epithelial cells, and ensure the smooth progress of mucociliary clearance and mucosal barrier homeostasis [25]. With its unique composition and functions, the respiratory mucus layer plays an irreplaceable role as a physical and immune barrier in the fight against pathogens, safeguarding the health of the respiratory tract. Targeting the barrier characteristics of the mucus layer, the core strategy is to balance “mucus penetration” and “mucosal retention”, and adjuvants/carriers need to possess the core capability of balancing mucus penetration and retention: on the one hand, optimizing physicochemical properties (e.g., charge and hydrophilicity) to reduce mucin binding, and on the other hand, maintaining appropriate adhesion after penetration to ensure effective retention at the epithelial surface—all while avoiding interference with normal mucus secretion and MCC function—defined as the coordinated interplay between three key components (the mucus layer, cilia, and airway epithelial cells) to achieve efficient physical clearance and mucosal homeostasis. The core synergy mechanism of the mucociliary clearance (MCC) system lies in the “capture-propulsion-regulation” closed loop formed by the mucus layer, cilia, and airway epithelial cells: the mucus layer traps pathogens, foreign particles, and vaccine components via a mucin-based gel; airway epithelial cells regulate mucus hydration through ion channels to adapt to ciliary beating and secrete immune-active molecules to optimize clearance efficiency; cilia generate directional shear force with coordinated rhythms to propel mucus loaded with foreign substances toward the pharynx for excretion. The dynamic collaboration between these three components not only ensures physical clearance efficiency but also maintains mucosal homeostasis, directly determining the residence time of intranasal vaccine components. It also requires vaccine adjuvants/carriers to resist rapid clearance without disrupting this synergistic operation, thereby balancing immunogenicity and biocompatibility. This synergy is highly relevant and critical to intranasal vaccine development: it directly governs the residence time of vaccine antigens and adjuvants in the nasal cavity, as the MCC system’s efficient coordinated clearance can rapidly eliminate unmodified vaccine components, limiting immune activation. Moreover, disrupting this synergistic function (e.g., inhibiting ciliary beating or altering mucus composition) may cause mucosal irritation, inflammation, or impaired defense, leading to safety risks. Thus, ideal intranasal adjuvants must be rationally designed to adapt to or moderately modulate this synergistic mechanism—resisting rapid clearance while preserving the MCC system’s normal physiological function—to balance immunogenicity and biocompatibility [17,26].

### 2.3. Innate Immune Cells of the Respiratory Mucosa

The respiratory mucosal innate immune system serves as the first line of defense against inhaled pathogens, consisting not only of professional innate immune cells (e.g., macrophages, dendritic cells, and mast cells) but also innate lymphoid cells (ILCs, particularly type 2 ILCs) and natural killer (NK) cells. In rodents, nasal-associated lymphoid tissue (NALT) acts as a key inductive site for mucosal immune responses, whereas in non-rodent species such as humans and non-human primates, the palatine tonsils, adenoids and nasopharyngeal tonsils are the major mucosal lymphoid inductive tissues. These tissues mediate the initiation of antigen-specific immune responses following intranasal immunization and thus play an indispensable role in nasal mucosal immunity. In addition, both NALT and tonsillar tissues are rich in adaptive immune cells including B lymphocytes and T lymphocytes, which interact closely with subsets of innate immune cells to collectively form a coordinated, layered mucosal immune defense network [27]. As professional antigen-presenting cells (APCs), DCs can activate naive T cells to initiate adaptive immune responses; CD4^+^ T cells stimulated by DCs further activate naive B cells, which undergo isotype class switching to form committed B cells capable of secreting specific IgA antibodies—these cells highly express the mucosal homing receptor CCR10 and migrate from lymph nodes into the bloodstream [28]. In addition to the aforementioned immune cells, mast cells play a crucial role in nasal mucosal immunity: they can rapidly respond to external stimuli, releasing antimicrobial peptides and reactive oxygen species to directly kill bacteria, and secrete cytokines such as IL-4, IL-10, and IL-13 to promote the differentiation of naive T cells into Th2 cells and enhance humoral immune responses; they can also secrete CCL20 to recruit DC precursors from the blood to inflammatory sites and promote tissue edema, facilitating DC migration to draining lymph nodes, thereby linking innate immunity with adaptive immunity [29]. These immune cells synergistically construct the nasal intended meaningmucosal immune defense line. Simple antigen immunization, by contrast, is difficult to elicit an immune response of sufficient intensity. Therefore, small-molecule immune adjuvants have emerged as a research focus for intranasal vaccine development. Given the difficulty of simple antigen immunization in eliciting sufficient immune responses, these adjuvants offer distinct advantages: they can enhance immune responses through mechanisms such as promoting antigen presentation by antigen-presenting cells (APCs) and activating mucosal immune cell activity. Moreover, their small molecular weight facilitates penetration of the respiratory mucosal barrier, allowing them to efficiently reach target immune cells and thereby improve the body’s resistance to pathogens.

In summary, the nasal mucosa constructs an effective frontline defense through its sophisticated physical structures (such as the MCC system and the tightly junctional epithelial barrier) and an active network of immune cells. However, these protective mechanisms, designed to defend against pathogens, conversely become major obstacles for the effective delivery of vaccines and the elicitation of immune responses: antigens may be rapidly cleared by mucus, degraded by enzymes, or struggle to traverse the epithelial barrier to reach areas populated by immune cells.

It is precisely these challenges that underscore the critical role of adjuvants and intelligent delivery systems in the design of intranasal vaccines. An ideal intranasal vaccine adjuvant is expected to not only enhance the immunogenicity of the antigen but also be capable of overcoming the aforementioned barriers—whether by increasing mucoadhesion to resist clearance, by temporarily and reversibly modulating epithelial permeability to facilitate antigen uptake, or by directly targeting and activating underlying innate immune cells. Toll-like receptor (TLR) agonists, which will be reviewed in detail in the following section, represent a key class of such adjuvant strategies. By mimicking pathogen-associated molecular patterns (PAMPs), they can precisely activate TLR signaling pathways in APCs (e.g., DCs), thereby initiating robust mucosal and systemic immune responses and offering an effective strategy for overcoming barrier limitations.

## 3. Pattern Recognition Receptor Agonist

Given that the respiratory mucosal barrier poses multiple obstacles to vaccine delivery, how to precisely activate mucosal immune responses has become the key. Pattern recognition receptors (PRRs), acting as signal switches on the surface of immune cells, provide a core solution to this problem. Based on their ligand recognition and structural characteristics, PRRs can be divided into several types: TLRs, the cGAS-STING pathway, C-type lectin receptors, nucleotide-binding oligomerization domain (NOD)-like receptors (NLRs), retinoic acid-inducible gene I (RIG-I)-like receptors (RLRs), and absent in melanoma 2 (AIM2)-like receptors (ALRs) [30].

### 3.1. TLR Agonist

TLRs are the most well-characterized pattern recognition receptors (PRRs)—a class of evolutionarily conserved innate immune receptors that recognize pathogen-associated molecular patterns (PAMPs) from microorganisms. Compared with other PRRs, TLRs have broader ligand recognition properties. The structure of TLR ligands consists of lipids, polysaccharides, proteins, and nucleic acids. Thirteen TLRs (TLR1–TLR13) have been identified in mammals, among which TLR1–TLR10 are expressed in humans. These TLRs are type I transmembrane proteins, and their structural features include an extracellular domain, a transmembrane region, and a cytoplasmic Toll/IL-1 receptor (TIR) domain. TLR agonists bind to the extracellular domain, and the TIR domain interacts with downstream signaling molecules to initiate cellular signal transduction. TLRs are localized in different cellular compartments and can recognize PAMPs from a variety of microorganisms [31]. As adjuvants, TLR agonists have the advantages of more targeted immune activation, precise activation of specific signaling pathways in immune cells, and efficient induction of immune responses. Additionally, some TLR agonists can regulate the type of immune response and modulate Th1 and Th2 immune responses (Table 1).

#### 3.1.1. Agonists for Cell Membrane-Localized TLRs

##### TLR5 Receptor Agonist: Flagellin (FliC)

FliC is the main structural component of flagella and a highly conserved protein that is recognized by TLR5 in the host. As a potent immune activator, FliC stimulates a variety of biological effects, mediating innate inflammatory responses and the development of adaptive immunity. It can promote the activation and proliferation of local immune cells in the nasal mucosa, such as DCs and macrophages. After engulfing antigens, these cells can quickly transmit antigen information to lymphocytes, triggering specific immune responses, thereby effectively containing pathogens at the initial stage of invasion [46]. Studies have shown that FliC has the advantages of high efficiency, low toxicity, and ease of preparation and modification. Moreover, flagellin-based vaccines have demonstrated good immunogenicity in various disease models, making them capable of inducing the production of high levels of antigen-specific antibodies, including IgG1 and IgG2a subtypes, thus providing protection against pathogens [47].

Qian et al. [36] investigated the mucosal adjuvant activity of Salmonella FliC as a carrier in the EXP153-rFliC conjugate peptide. EXP153-rFliC is formed by conjugating a B-cell epitope peptide synthesized from Plasmodium falciparum export protein-153 (EXP153) with the recombinant flagellin (rFliC) of the Salmonella enterica serovar Typhimurium, which is expressed in Escherichia coli. Experiments showed that intranasal administration of EXP153-rFliC enhanced Th1-type responses but still predominantly induced Th2-type responses. Salmonella FliC is an effective mucosal adjuvant for its conjugated peptide, and intranasal administration of the EXP153-rFliC conjugate can induce systemic and mucosal EXP153-specific antibody responses in mice, providing a new approach for its application in conjugate vaccines.

Building on the demonstration that Salmonella FliC functions as a potent mucosal adjuvant for parasitic epitopes, researchers have extended its application to bacterial pathogens, exploring both fusion protein strategies and synergistic adjuvant combinations. Asadi Karam et al. [37] prepared a fusion protein by combining FliC with FimH (a protein expressed by uropathogenic Escherichia coli, UPEC), and used FliC and cholera toxin (CT) as adjuvants for intranasal immunization in mice to enhance protective immunity against UPEC antigens. Experiments showed that FliC, as an adjuvant, tended to enhance Th1 and Th2 responses against FimH, and intranasal immunization induced higher cellular and serum antibody responses than subcutaneous immunization. FliC was more immunogenic than mixed antigens and single antigens and could clear UPEC infection more effectively. Furthermore, the anti-FimH and anti-FliC IgG antibodies were induced by FimH. FliC fusion vaccine formulations persisted for a long time—at least 28 weeks after the first immunization and 24 weeks after the third immunization. CT, as another adjuvant, had a good synergistic effect with FliC, which could significantly enhance the anti-FimH and anti-FliC IgG responses of the vaccine formulation. In addition, the vaccine formulation containing CT could induce strong humoral and cellular immune responses, providing significant protection for the bladder and kidneys.

As a TLR5 agonist, FliC confers distinct advantages of high efficacy and low toxicity in mucosal immune activation. Nevertheless, single-target engagement is often constrained by certain limitations, such as skewed immune response polarization. In comparison, TLR4 agonists modulate immune responses via divergent signaling cascades, presenting a novel avenue to broaden the applicability of intranasal vaccines.

##### TLR4 Agonist: PS-G (Polysaccharide Purified from Ganoderma Lucidum)

PS-G is a TLR4 agonist that can induce DCs to secrete IL-12 via TLR4 activation. IL-12 plays a central role in linking innate and adaptive immunity, as it stimulates the growth and differentiation of T lymphocytes, promotes the proliferation and activation of Th1 cells, and enhances cellular immune responses [48]. A study investigated the role of PS-G as an adjuvant for enterovirus A71 (EV-A71) mucosal vaccines. Experiments demonstrated that intranasal immunization with the EV-A71 vaccine adjuvanted with PS-G could induce IgA antibody production in the respiratory tract and other mucosal sites, generating both mucosal and systemic immune responses. Furthermore, the vaccine could resist infection by different genotypes of EV-A71, exhibiting broad cross-protective effects with no obvious toxicity in mice [38].

As a naturally derived TLR4 agonist, PS-G exhibits outstanding performance in terms of safety and cross-protection. In contrast, synthetic or modified TLR4 agonists offer greater room for optimization in immune enhancement efficiency, with inulin acetate (InAc) serving as a typical representative of such novel TLR4 agonists.

##### Natural TLR4 Agonist: InAc

In addition, InAc, a plant-based polymer, has been shown to activate TLR4 and act as a vaccine adjuvant to elicit robust systemic immune responses. The authors evaluated the potential of InAc nanoparticles (InAc-NPs) as an intranasal vaccine delivery system to induce mucosal and systemic immune responses, with PLGA-NPs used as a control. Compared with PLGA-NPs, intranasal immunization with antigen-loaded InAc-NPs resulted in 65-fold and 19-fold increases in IgG1 and IgG2a titers, respectively. InAc-NPs also stimulated the secretion of sIgA in various mucosal sites, including NALTs, lungs, and intestines, and induced strong memory responses—indicating activation of humoral and cellular immunity—suggesting great potential as an adjuvant for intranasal vaccines [39].

##### TLR4 Agonist: PHAD (Phosphorylated HexaAcyl Disaccharide)

PHAD (Phosphorylated HexaAcyl Disaccharide) is a synthetic TLR4 agonist and an artificial analog of lipid A. Its structure closely mimics that of natural monophosphoryl lipid A (MPLA), enabling it to specifically activate the TLR4 signaling pathway and induce Th1-type immune responses. It features a well-defined structure and high batch-to-batch stability, often serving as a positive control in TLR4 adjuvant research. However, its immune activation efficacy is weaker than that of the BECC-series adjuvants, and it lacks the ability to balance Th1/Th2 responses.

Lederhofer et al. developed a respiratory syncytial virus (RSV) vaccine, replacing the naturally occurring MPLA—which features structural heterogeneity and high production difficulty—with 3D-PHAD^®^ (a core derivative of PHAD) as the TLR4 agonist adjuvant to create a 3D-PHAD^®^-adjuvanted RSV virosomal vaccine, which fully highlighted the core advantages of 3D-PHAD^®^. First, it is a well-defined, fully synthetic compound: as a synthetic active monomer of MPLA, it addresses the issues of complex composition and poor batch-to-batch stability associated with natural MPLA, making it more suitable for the large-scale production of vaccines. Second, it exhibits high adjuvant activity at a lower dosage: localized on the outer membrane of virosomes via the DMSO post-insertion method, a 3D-PHAD^®^-to-viral protein ratio of only 0.1 mg/mg induced titers of RSV-specific neutralizing antibodies and IgG antibodies comparable to those induced by the DCPC solubilization method at a ratio of 0.2 mg/mg, achieving highly efficient immune activation even with a 50% reduction in adjuvant dosage. Third, it has excellent formulation compatibility: it can be incorporated into the virosomal membrane through multiple methods without compromising the physicochemical stability of the vaccine. The prepared vaccine had a particle size of approximately 96.3 nm, and only slight reversible aggregation was observed after 300 days of storage at 4 °C, with both its structure and adjuvant activity remaining stable. This study confirmed that 3D-PHAD^®^, as an excellent synthetic alternative to MPLA, enables the successful preparation of RSV virosomal vaccines with high immunogenicity and long-term stability by virtue of its multiple advantages in structure, activity and formulation compatibility, thus providing a better option for the selection of adjuvants for respiratory vaccines [40].

##### TLR4 Agonist: GLA (Glucopyranosyl Lipid A)

GLA (Glucopyranosyl Lipid A) is a synthetic, non-toxic lipopolysaccharide analog and potent TLR4 agonist. It binds to TLR4 on immune cells like dendritic cells and macrophages, inducing cell maturation, upregulating co-stimulatory molecules, and promoting the secretion of cytokines such as IL-12 p70 to skew Th1-type immune responses. Synergizing with agonists like 3M-052, it enhances antigen-specific T/B cell responses and immune memory formation. As it is compatible with various antigens and delivery routes (e.g., intranasal, intramuscular), it is widely used as an adjuvant in anti-infective vaccines (especially mucosal vaccines) for influenza and amebiasis, establishing combined mucosal and systemic immunity at pathogen entry sites. With proven preclinical efficacy and ongoing clinical evaluations, it is a promising core adjuvant in vaccine development.

Voigt et al. developed a novel tuberculosis vaccine comprising the recombinant antigen ID93 and the liposomal adjuvant GLA-3M-052-LS to optimize BCG’s protective efficacy against *Mycobacterium tuberculosis* (Mtb). As core adjuvants, the TLR4 agonist GLA and the TLR7/8 agonist 3M-052 act synergistically—complementing pathways, amplifying signals, enhancing Th1-type cellular and humoral immunity, and meeting mucosal protection needs while avoiding single immune bias. In Mtb-susceptible CC004 mice, the vaccine, via a heterologous intramuscular–intranasal regimen, induced high levels of systemic ID93-specific IgG, bone marrow long-lived IgG-secreting cells, as well as pulmonary mucosal IgA and polyfunctional Th1-type CD4^+^ T cell responses, with an immunological score far superior to that of the traditional adjuvant GLA-SE. Mtb aerosol challenge experiments showed that it effectively maintained mouse body weight, reduced pulmonary bacterial load, and decreased the lung granuloma area and pro-inflammatory cell infiltration, with its protective efficacy exceeding that of BCG alone or homologous intramuscular immunization. These characteristics confirm that the adjuvant system synergistically activates innate immunity to enhance both mucosal and systemic immunity, providing a highly effective candidate for tuberculosis vaccine development [41].

Murphy et al. developed a novel intranasal vaccine against amebiasis, whose liposomal adjuvant is composed of the synthetic TLR4 ligand GLA and the TLR7/8 ligand 3M-052. As the core immunopotentiating component, GLA plays a pivotal role in boosting immune responses by activating the TLR4 pathway and exerts a remarkable synergistic effect with 3M-052 to optimize the vaccine’s immunogenicity—this adjuvant combination had been verified to induce robust and durable immune responses as well as effective protective efficacy in a murine model of amebic colitis in previous studies. The research team conducted in-depth in vitro delivery characterization of the vaccine with the Teleflex MAD Nasal™ device, and the results demonstrated that this vaccine–device combination realized highly efficient targeted deposition in both adult and infant nasal cavity models, with the vaccine’s lung penetration score being extremely low and well below the limit of quantification. These findings fully confirmed that the GLA-containing adjuvant system can effectively guarantee the immunogenicity of the amebiasis vaccine and enable the vaccine to be efficiently delivered to the target mucosal sites in the nasal cavity of both adults and infants, laying a solid foundation for the subsequent research, development and clinical translation of the amebiasis vaccine and also providing valuable references for the adjuvant selection and delivery system optimization of other mucosal vaccines [42].

##### TLR4 Agonist: BECC (Bacterial Enzymatic Combinatorial Chemistry)

BECC (Bacterial Enzymatic Combinatorial Chemistry) is a biosynthetic technology for developing vaccine adjuvants, with core products including TLR4 agonists such as BECC438 and BECC470—obtained by modifying the bacterial lipid A backbone, featuring well-defined structures (diphosphorylated and monophosphorylated hexaacyl structures, respectively) and enabling large-scale production at low cost. Its key advantage lies in inducing balanced Th1/Th2 immune responses, with low endotoxin toxicity and superior immune activation efficacy compared to traditional adjuvants. Compatible with various immunization regimens, it represents an important research direction for broad-spectrum and high-efficacy vaccine adjuvants [43].

In their study, Robert E. Haupt et al. compared the immunological efficacy of novel BECC-series TLR4 agonists (BECC438 and BECC470) with that of the traditional adjuvant aluminum hydroxide (alum) and the synthetic TLR4 agonist PHAD (Phosphorylated HexaAcyl Disaccharide), clarifying the core advantages of the BECC series: Derived from the modification of the bacterial lipid A backbone via Bacterial Enzymatic Combinatorial Chemistry (BECC) technology, these agonists enable large-scale production at low cost, exhibit lower endotoxin toxicity than Escherichia coli lipopolysaccharides (LPSs), and possess superior innate immune activation capacity compared to PHAD. Notably, they induce a balanced Th1/Th2 immune response (simultaneously elevating IgG1 and IgG2a antibody titers), whereas alum favors a Th2 response, and PHAD leans toward a Th1 response. Using recombinant hemagglutinin (rHA) as the antigen, the BECC-adjuvanted groups demonstrated significant advantages in both prime-boost and prime-only immunization regimens: total serum IgG antibody titers were substantially higher than those in other adjuvanted groups, and the protective efficacy against homologous (NL/09 H1N1) and heterologous (Sing/2015 H1N1) influenza A virus infections was optimal—mice showed only ~7% weight loss, undetectable viral loads in lung tissues, and mild pulmonary inflammation. Additionally, the BECC agonists exhibited obvious antigen/adjuvant-sparing effects (reducing the antigen dosage by over 7-fold and the adjuvant dosage by 10-fold), providing crucial support for the development of influenza vaccines that require no annual updates, are effective with a single dose, and offer broad-spectrum cross-protection [43].

#### 3.1.2. Agonists for Endosome-Localized TLRs

##### TLR9 Agonist: CpG

The aforementioned cell membrane-localized TLR agonists exert their effects primarily by activating receptors on the cell membrane, whereas endosome/cytoplasm-localized TLR agonists can target intracellular signaling pathways, forming a complementary immune activation pattern. Among these, the TLR9 agonist CpG has been the most extensively studied.

CpG oligodeoxynucleotides (ODNs) are TLR9 agonists that can induce innate immune responses by activating TLR9. TLR9 is mainly expressed in the endosomes of immune cells such as plasmacytoid DCs and B cells. After pathogen-derived unmethylated CpG DNA enters the endosome, it specifically binds to and activates TLR9, thereby inducing a predominantly Th1-type immune response [49].

Lin et al. [32] evaluated the role of CpG as an intranasal adjuvant in the mucosal vaccine against EV71-associated hand, foot, and mouth disease (HFMD). The experimental results showed that CpG, as an intranasal adjuvant, could induce systemic and mucosal EV71-specific antibody responses. The titers of EV71-specific IgG and IgA in serum, nasal washes, bronchoalveolar lavage fluids, and feces of the EV71 + CpG group were significantly higher than those in other groups. Additionally, the production of EV71-specific IgG2a was significantly increased, indicating a bias toward Th1-type responses. Furthermore, the intranasally immunized EV71 vaccine induced Th17-type immune responses, with increased EV71-specific IgA responses and IL-17-secreting T-cell responses induced by intranasal immunization.

Building on the promising performance of CpG as a mucosal adjuvant for viral vaccines, researchers have extended its application to emerging respiratory pathogens, with notable success in SARS-CoV-2 vaccine development. Russo et al. [33] developed an intranasal vaccine formulation against SARS-CoV-2 using a TLR9 agonist as an adjuvant. It consists of the cationic liposome carrier 1,2-dioleoyl-3-trimethylammonium-propane (DOTAP), the SARS-CoV-2 trimeric spike protein, and CpG ODNM. Experiments showed that intranasal and subcutaneous vaccination had equivalent efficacy in protecting mice against SARS-CoV-2 infection; however, intranasal vaccination was more effective in clearing lung viruses, inducing neutralizing antibodies and sIgA antibodies, and preventing neuroinflammation. Meanwhile, the formulation was shown to protect mice against infection by multiple SARS-CoV-2 variants. In vitro and in vivo experiments demonstrated no toxicity following intranasal administration of this vaccine formulation.

Beyond single-pathogen applications, studies have also explored the synergistic potential of CpG with other bioactive molecules to enhance its preventive and therapeutic efficacy against influenza virus—a classic respiratory pathogen with a high mutation rate. Ontiveros-Padilla et al. [34] investigated a nanoparticle adjuvant system composed of M7 and CpG for influenza vaccines. Mastoparan-7 (M7) is a cationic peptide derived from wasp venom with strong adjuvant activity. When used with antigens, M7 can activate G protein signaling pathways, leading to mast cell degranulation and activation via the mast cell membrane receptor MrgprX2. Experimental results showed that the serum IgG and IgM titers in the M7-CpG NP group were significantly higher than those in the single-adjuvant groups. The levels of IgG1, IgG2c, and IgA in serum, nasal cavity, and feces were also higher; the number of IL-2- and IFN-γ-expressing cells in splenocytes increased, and IFN-γ levels were elevated. IL-4 levels were lower than those in the M7 group, and the frequency of central and effector memory CD4^+^ T cells in draining lymph nodes (dLNs) increased. Moreover, compared with the CpG single-adjuvant group, the survival rate of mice infected with the influenza virus in the M7-CpG NP group significantly increased to 100%.

Regarding the application of CpG as an adjuvant for intranasal SARS-CoV-2 vaccines, a study compared intranasal administration of formulations combining the SARS-CoV-2 spike protein (S protein) receptor-binding domain (RBD) or S1 protein with CpG oligonucleotides and the aluminum hydroxide (alum) adjuvant, respectively. Compared with the traditional alum adjuvant, CpG ODN had the ability to activate Th1-type immune responses and exhibited strong advantages in inducing mucosal immunity. In terms of neutralizing antibody titers, the intranasally vaccinated S1-CpG group had significantly higher neutralizing antibody titers in serum and BAL than the alum adjuvant group [35].

It is worth noting that CpG, as an intranasal adjuvant, poses potential toxicity risks that require focused attention in applications. High doses of CpG ODNs (≥50 μg/dose) can induce nasal mucosal epithelial cell necrosis in mice, with a mucosal damage rate as high as 20%. However, reducing the dose to mitigate toxicity may compromise immunogenicity—for instance, decreasing the CpG dose to 10 μg/dose results in a 40% reduction in the titer of secretory immunoglobulin A (sIgA) in nasal lavage fluid [32]. This dose-dependent trade-off between toxicity and immunogenicity is a key challenge that must be addressed when using CpG in intranasal vaccines. Additionally, CpG may induce excessive cytokine release, leading to the risk of systemic inflammation, which often prompts regulatory authorities to require additional pharmacodynamic studies to rule out potential safety hazards. Therefore, in the design of intranasal vaccines, strategies such as precise dose regulation and optimization of delivery systems (e.g., combination with liposomes or nanoparticles) should be adopted to maximize the retention of its immune-enhancing activity while minimizing mucosal toxicity and systemic safety risks, thereby facilitating its clinical translation.

##### TLR3 and RIG-I Agonist: Poly I:C

Polyinosinic-polycytidylic acid (Poly I:C), which is structurally similar to dsRNA, is a typical synthetic TLR3 agonist. Studies have shown that in addition to TLR3, Poly I:C can also activate RIG-I and melanoma differentiation-associated protein 5 (MDA5). Via the TRIF-mediated signaling pathway, Poly I:C activates transcription factors such as IRF1, IRF7, IRF3, and NF-κB; induces the expression of type I interferons, pro-inflammatory cytokines, and antigen presentation-related molecules; promotes DC maturation; and enhances antigen-specific T-cell responses. However, Poly I:C has limitations including limited solubility, difficulty in re-forming double strands, poor stability, susceptibility to degradation, short half-life, and a tendency to cause severe side effects such as renal failure, hypotensive shock, and thrombosis [50].

### 3.2. cGAS2-STING Agonist-Based Vaccine Adjuvants

The cGAS-STING pathway serves as a central signaling axis of innate immunity, which senses aberrant cytoplasmic double-stranded DNA (dsDNA) derived from pathogenic invaders or host cells. Upon activation, it triggers the secretion of type I interferons and proinflammatory cytokines via downstream signaling cascades, acting as a critical bridge that connects innate and adaptive immunity and exhibiting a bias toward inducing Th1-type cellular immunity against intracellular pathogens [51,52]. STING agonists, including the synthetic agonist ADU-S100 and cGAMP (cyclic GMP-AMP), can selectively activate this pathway, potently elicit effector T-cell responses, and thus represent a promising research direction for the development of mucosal vaccine adjuvants. They possess prominent immunostimulatory advantages in vaccines targeting intracellular infections, making them a valuable complement to TLR agonists in mucosal immune activation.

#### 3.2.1. Synthetic Agonist: ADU-S100

Taylor B Poston et al. investigated a CPAF-based vaccine against *Chlamydia trachomatis* genital infection, focusing on the intranasal adjuvant activity of ADU-S100, a synthetic cGAS-STING agonist. This study provided the first direct evidence for applying the cGAS-STING pathway in chlamydial mucosal vaccines. All formulations showed favorable mucosal safety. Activation of the cGAS-STING pathway was identified as critical for protective anti-chlamydial immunity: ADU-S100 alone strongly induced CD4 T cell-dominated cellular immunity and Th1-biased humoral immunity, matching the immune requirements for intracellular chlamydial control. It markedly reduced chlamydial burden in mice by 1.5 log orders, accelerated infection clearance, and decreased hydrosalpinx. No synergistic effect was detected with other adjuvants, supporting the idea that specific cGAS-STING activation delivers optimal immune stimulation for mucosal defense. This work highlights the core role of the pathway in eliciting targeted cellular immunity, fills a gap in chlamydial vaccine research, and supports the clinical development of cGAS-STING agonists as intranasal adjuvants for mucosal vaccines against intracellular pathogens [53].

#### 3.2.2. Synthetic Agonists: RR-CDG and ML-RR-cGAMP

Van Dis et al. investigated the application of STING-activating cyclic dinucleotides (CDNs) as adjuvants in *Mycobacterium tuberculosis* vaccines. The results demonstrated that protein subunit vaccines containing CDNs (such as RR-CDG and ML-RR-cGAMP), with the 5Ag fusion protein as the antigen (comprising *M. tuberculosis* antigens including Ag85B and ESAT-6), exhibit significant protective efficacy: When administered subcutaneously, their protective effect against *M. tuberculosis* infection in mice is comparable to that of the live-attenuated Bacillus Calmette–Guérin (BCG) vaccine. This protection is STING-dependent but independent of type I interferon signaling, and it can induce the CXCR3^+^KLRG1^−^CD4^+^ T-cell subset that homes to the lung parenchyma. Intranasal administration yields even better outcomes, eliciting dual Th1 and Th17 immune responses (with the Th17 response associated with enhanced protection). When used alone, it achieves 1.5–2 log orders of protection, and its protective efficacy is further improved when used as a booster for the BCG vaccine. Additionally, ML-RR-cGAMP, a STING agonist suitable for humans, can induce similar protective effects when administered intranasally. These findings confirm that CDNs, as adjuvants, can stimulate targeted T-cell responses through mucosal or subcutaneous delivery routes, providing long-lasting protection against *M. tuberculosis* infection and offering a novel strategy for the development of tuberculosis vaccines [54].

#### 3.2.3. Natural Agonist: cGAMP

Wang et al. constructed pulmonary surfactant-biomimetic nanoparticles (PS-GAMP) encapsulating the STING natural agonist cGAMP, which were intranasally co-administered with inactivated influenza vaccines. Mechanistically, PS-GAMP is internalized by alveolar macrophages (AMs) via pulmonary surfactant proteins (SP-A/SP-D), releasing cGAMP that is then transferred to alveolar epithelial cells (AECs) through gap junctions. This dual mechanism activates the cGAS-STING pathway in both cell types, inducing type I interferons, GM-CSF, and other cytokines to recruit/mature CD11b^+^ dendritic cells, thereby potently eliciting influenza-specific CD8^+^ T-cell responses (including lung-resident memory T cells), mucosal IgA, and systemic IgG. The key advantage is as follows: a single intranasal immunization confers cross-protection against heterosubtypic influenza viruses (H1N1, H3N2, H5N1, and H7N9) within 2 days, with protection lasting up to 6 months, while avoiding excessive inflammation and showing excellent biocompatibility. This study confirmed that STING activation in AECs and gap junction-mediated cGAMP transport are critical for the pathway’s adjuvant effect, providing important support for developing broad-spectrum influenza vaccines [55].

In addition, to address the low immunogenicity of inactivated H7N9 vaccines observed in clinical trials, a study utilized cGAMP, a natural agonist of STING, as a mucosal adjuvant for intranasal immunization in combination with inactivated whole-virus H7N9 vaccines. Following intranasal delivery, cGAMP activates the cGAS-STING pathway in antigen-presenting cells (e.g., dendritic cells and macrophages) and respiratory mucosal cells, triggering downstream IRF3/NF-κB signaling cascades to induce the secretion of molecules such as type I interferons, IFN-γ, and granzyme B, thereby potently amplifying immune responses. This strategy demonstrated remarkable efficacy: A single intranasal immunization achieved 100% protection in mice challenged with a high lethal dose (40 × LD_50_) of the homologous H7N9 virus, accompanied by a significant dose-sparing effect. It also induced nucleoprotein-specific CD4^+^ and CD8^+^ T-cell responses, endowing mice with cross-protection against heterosubtypic viruses including H1N1, H3N2, and H9N2. This provides a crucial approach for the adjuvant optimization of low-immunogenicity influenza vaccines [56].

An et al. developed a single-dose intranasal subunit vaccine against SARS-CoV-2, using lyophilized trimeric and monomeric spike proteins as antigens and liposomally encapsulated 2′3′-cGAMP, a STING agonist, as the adjuvant. The vaccine formulation had good stability and showed no toxicity upon intranasal administration. In BALB/c mouse models, a single immunization induced comprehensive and durable systemic immunity and respiratory mucosal immunity. Systemically, high titers of neutralizing anti-spike IgG were detected in serum, and spike-specific T-cell responses were observed in the spleen and lung tissues. Mucosally, anti-spike IgA was produced in the nasal and lung tissues, and IgA-secreting B cells were present in the spleen. Single-cell RNA sequencing confirmed that nasal-associated lymphoid tissue served as the core immune inductive site of the vaccine, where the germinal center-like coordinated activation of B and T cells sustained the persistence of mucosal immunity. Characterized by non-invasive and easy administration, the vaccine required no ultra-low-temperature storage or transportation. It could build an immune barrier in the nasal cavity, the initial site of viral invasion, making up for the deficiency of conventional intramuscular vaccines that lack mucosal immunity and cannot effectively block viral transmission, thus establishing a novel platform for the development of respiratory viral vaccines. Further studies are needed to verify its sterile immune effect and immune persistence in higher animal models, as well as to optimize the vaccine antigen against viral variants [57].

Batty et al. developed a circular ten-nozzle multiplex electrospray apparatus for the high-efficiency preparation of acetalated dextran-encapsulated cGAMP microparticles, a potent STING agonist, with productivity an order of magnitude higher than single-head electrospray while retaining consistent physicochemical properties. In vitro, the microparticles strongly stimulated murine bone marrow-derived dendritic cells to secrete IFN-β, activating the STING pathway far more effectively than soluble cGAMP and at comparable levels with single-head-prepared microparticles. In vivo, when used as an adjuvant combined with the influenza COBRA HA Y2 antigen in C57BL/6 mice, they induced anti-HA IgG titers comparable to those of Addavax and a more robust Th1-type immune response with high IgG2c levels, overcoming the Th2 bias of traditional adjuvants, and no significant difference in adjuvant activity was found between multiplex and single-head microparticles. The study showed that multiplex electrospray enables the scalable production of cGAMP microparticle adjuvants with excellent STING pathway activation capacity, which can elicit balanced and potent immune responses for influenza subunit vaccines, laying a solid foundation for the large-scale manufacturing and clinical translation of this STING agonist adjuvant and further expanding the application of STING-activating cGAMP in the development of anti-infective vaccines [58].

#### 3.2.4. Natural Agonist: c-di-AMP

Cyclic diadenosine monophosphate (c-di-AMP), a bacterial-derived second messenger, serves as a core activator of the cGAS-STING pathway and a key adjuvant in the intranasal vaccine against *Trypanosoma cruzi* in this study, exerting remarkable immune-enhancing and anti-infection protective effects when combined with the trans-sialidase (TS) antigen derived from Lactococcus lactis. c-di-AMP can be recognized and bound by cyclic GMP-AMP synthase (cGAS) in host cells, thereby activating the stimulator of the interferon gene (STING) pathway and initiating the efficient transition from innate immunity to adaptive immunity [59].

Pacini et al. immunized BALB/c mice intranasally three times at 2-week intervals with the TS+c-di-AMP formulation. Compared with TS alone, the ISPA adjuvant combination, and the saline control, this formulation significantly induced immune responses: plasma TS-specific IgG2a titers and the IgG2a/IgG1 ratio were markedly increased, and nasal mucosal secretory IgA (sIgA) expression was strongly enhanced. Plasma antibodies from this group effectively inhibited *Trypanosoma cruzi* invasion of host cells in vitro. TS+c-di-AMP activated CD8^+^ T cells and promoted IFN-γ secretion, significantly increasing CD8^+^ IFN-γ^+^ T cells at levels similar to those in the ISPA group and much higher than those in controls. In the acute oral *T. cruzi* challenge, only the TS+c-di-AMP group effectively controlled parasitemia; reduced the parasite burden in the heart, muscle, and intestine; and alleviated tissue injury, clinical symptoms, and acute myocarditis. These results confirm that c-di-AMP acts as a vaccine adjuvant by activating the cGAS-STING pathway to coordinately trigger systemic humoral immunity, mucosal local immunity, and Th1-type cellular immunity, thereby conferring potent protection against acute *T. cruzi* infection and highlighting the central role of the cGAS-STING pathway in mucosal vaccine development [60].

## 4. Cytokine Adjuvants

While TLR agonists provide clear immune activation targets for intranasal vaccines by precisely recognizing PAMPs, the immune effects of single-target activation remain limited. Additionally, some TLR agonists face the challenge of balancing dose-dependent toxicity and mucosal irritation. In contrast, cytokine adjuvants can directly regulate immune cell differentiation and cytokine secretion profiles, specifically compensating for the shortcomings of TLR agonists in regulating immune response types and inducing immune memory. Nevertheless, single-target engagement is often constrained by certain limitations, such as skewed immune response polarization.

Early studies have found that when IL-1 family cytokines are used as mucosal vaccine adjuvants and administered intranasally in combination with influenza virus vaccine antigens, they can induce a two-layer protective immunity and may be applied in antiviral intranasal vaccines [61].

Currently, type I interferons, as adjuvants for intranasal inactivated influenza vaccines, have successfully completed Phase I clinical trials (NCT 00436046). Studies have revealed that the IL-33 and type 2 innate lymphoid cell (ILC2) axis can enhance influenza vaccines. Intranasal co-administration of IL-33 and the Fluzone influenza vaccine significantly improved the survival rate of mice infected with homologous CA04 and heterologous PR8 viruses, reduced the degree of weight loss and lung pathological damage, and enhanced viral clearance ability; this cross-protective effect can last for at least 24 weeks. IL-33-activated ILC2s are a key early event in establishing mucosal humoral immunity and are crucial for cross-protection against influenza viruses. This finding challenges traditional vaccine immunity concepts [62].

During the COVID-19 pandemic, IL-15 has been proposed as a nasal mucosal adjuvant in the development of novel SARS-CoV-2 vaccines. A study used IL-15 and S1 protein nanoparticles with TLR agonists as boosters for aluminum-adjuvanted vaccines and found that mucosal vaccines cleared viruses introduced into the upper respiratory tract more efficiently and could induce trained innate immunity [50]. Other cytokines, such as those from the GM-CSF family, the TNF family, as well as IL-2, IL-4, IL-5, IL-6, IL-10, IL-12, and IL-18, have also been used as mucosal adjuvants to enhance sIgA and systemic immune responses, and they have been investigated as candidate adjuvants for intranasal vaccines. Overall, cell factor adjuvants have been partially studied as mucosal adjuvants for inactivated vaccines and subunit vaccines, with preliminary research results reported in [63].

## 5. Carrier Adjuvants

**Figure 2:**
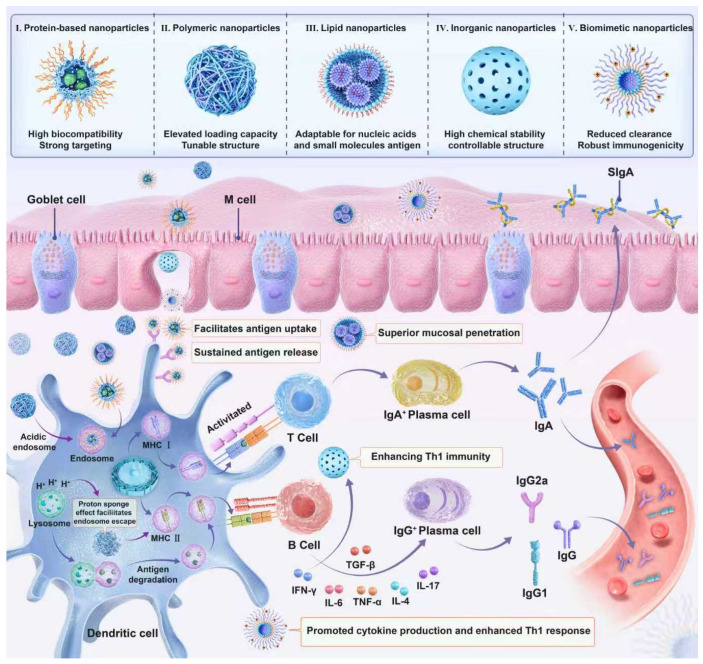
Advantages and disadvantages of nanoparticle-based mucosal delivery systems for common intranasal vaccine adjuvants.

While TLR and cytokine adjuvants have demonstrated promising potential to boost mucosal and systemic immune responses by modulating immune cell activation and cytokine cascades, their application in intranasal vaccines is still constrained by challenges such as short in vivo half-lives, potential off-target inflammatory effects, and difficulties in targeted delivery to nasal mucosal immune sites. In contrast, carrier adjuvants emerge as a robust and complementary strategy that addresses these limitations effectively. Acting as both antigen delivery vehicles and immune potentiators, carrier adjuvants not only protect labile antigens (e.g., proteins and nucleic acids) from degradation in the harsh nasal microenvironment but also enhance antigen uptake by mucosal immune cells through their tailored size, surface charge, and morphology. Moreover, the rational design of carrier adjuvants enables co-delivery of antigens and immunostimulants (e.g., TLR agonists and cytokines) in a spatiotemporally controlled manner, maximizing synergistic immune activation while minimizing systemic toxicity. This dual functionality makes carrier adjuvants an indispensable platform for advancing the development of next-generation intranasal vaccines with high efficacy and safety profiles (Figure 2).

### 5.1. Synthetic Biodegradable Polymeric Nanoparticles

#### 5.1.1. Polycaprolactone (PCL)-Based Nanoparticles

PCL is a biodegradable polymer material with immunomodulatory effects, showing broad application prospects in the field of vaccine development. PCL microparticles exhibit regular spherical morphology and smooth surfaces, which facilitate their adhesion and diffusion on the nasal mucosal surface, increase contact opportunities with mucosal epithelial cells, and thus promote their uptake into the body. The smooth surface may reduce the aggregation of microparticles on the mucosal surface, allowing more uniform distribution of microparticles on the mucosa and improving vaccine delivery efficiency. However, PCL has certain disadvantages such as poor hydrophilicity and an excessively slow degradation rate; thus, it is commonly combined with other carrier polymers in relevant studies.

Li et al. [64] obtained polycaprolactone–polyethylene glycol (PCL-PEG) and polycaprolactone–polyethyleneimine (PCL-PEI) through chemical synthesis; both are amphiphilic block copolymers that can self-assemble into micellar structures in aqueous environments. The study found that different polymer mass ratios had a significant impact on the efficacy of the formulation. An appropriate mass ratio could effectively enhance nasal residence time and cellular uptake, induce DC maturation, and improve cellular and humoral immune effects.

Çoban et al. [65] developed a biodegradable microparticle mucosal vaccine delivery system for diphtheria toxoid (DT) using materials such as poly(lactic-co-glycolic acid) (PLGA), PCL, polyvinyl alcohol (PVA), and DT and prepared DT-loaded microparticles by the double emulsion (W/O/W) solvent evaporation method. Experiments showed that intranasal administration of DT-loaded microparticles could induce systemic immune responses; when administered intranasally as a booster dose after subcutaneous inoculation, IgG titers increased significantly. PCL polymers induced a stronger immune response than PLGA polymers (*p* < 0.05), and PCL polymers alone had a stronger adjuvant effect, while PLGA polymers had no adjuvant effect when used intranasally. However, the in vitro active release ability of PCL was weaker than that of PLGA.

In addition to being used as a carrier for intranasal vaccines, PCL also exhibits the effect of intranasal administration for brain-targeted delivery. Recently, Deshmukh et al. [66] developed paclitaxel (PTX)-loaded PCL nanoparticles (NPs), mainly evaluating the brain-targeted delivery effect of these nanoparticles via intranasal administration. Ascorbic acid (AA) was conjugated to the surface of the nanoparticles as a targeting ligand. Since AA can cross the blood–brain barrier (BBB) through the SVCT2 transporter, modifying it on the surface of PTX-PNPs promoted the transport of nanoparticles to the brain via sodium–vitamin C cotransporter 2 (SVCT2), significantly increasing drug accumulation in the brain. In vitro drug release studies showed that the formulation released drugs faster in an acidic environment (e.g., tumor microenvironment and pH 5.5), which is beneficial for improving cytotoxicity against brain tumor cells. The sustained release property allows the drug to maintain a high concentration in the brain for a long time, enhancing the therapeutic effect on brain diseases.

#### 5.1.2. Poly(lactic-co-glycolic acid) (PLGA)-Based Nanoparticles

**Figure 3:**
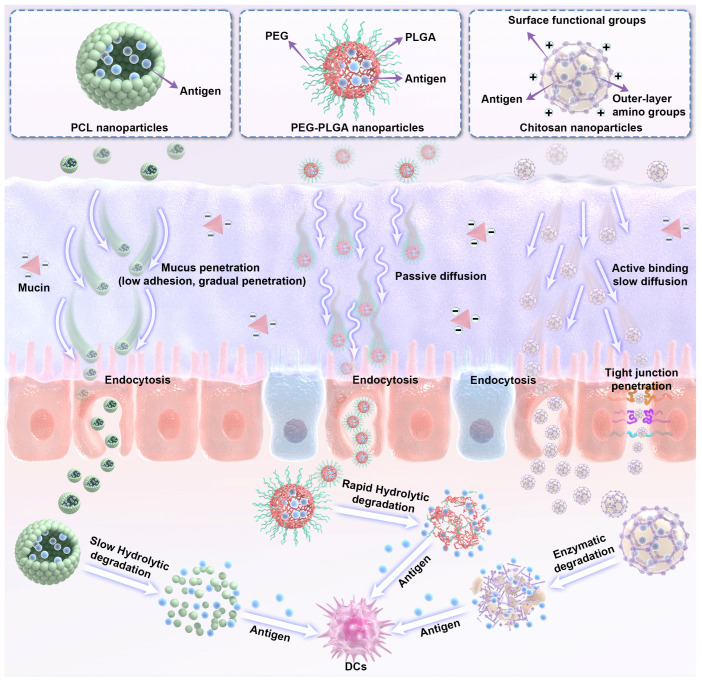
Differences in mucosal penetration and antigen release among three common polymeric nanoparticle adjuvant carriers.

While PCL excels in adjuvant activity and structural stability, its limited hydrophilicity and slow degradation drive the need for alternative synthetic polymers, with PLGA emerging as a clinically validated counterpart. PLGA is a polyester copolymer of polylactide (PLA) and polyglycolide (PGA); as a synthetic polymer, it undergoes ester bond hydrolysis in the human body to form lactic acid and glycolic acid, which are ultimately metabolized and eliminated as carbon dioxide and water. Approved by the FDA for vaccine and drug delivery as well as tissue engineering, PLGA offers exceptional biocompatibility, with tunable biodegradability via adjustments to its composition, molecular weight, and chemical structure (Figure 3) [67].

Kabiri et al. [68] prepared human T-cell leukemia virus type 1 (HTLV-1) fusion epitope-loaded PLGA nanoparticles. HTLV-1 is a carcinogenic retrovirus that infects a large number of people worldwide, causing diseases such as adult T-cell leukemia and tropical spastic paraparesis. The authors prepared chimeric and CpG ODN-loaded PLGA nanospheres using the double emulsion (W/O/W) solvent evaporation technique. The HTLV-1 fusion epitope is a multi-epitope chimera formed by linking immunodominant epitopes such as Tax, env (gp21 and gp46), and gag (p19), and it is designed as a vaccine component capable of effectively inducing immune responses against HTLV-1.

Experiments showed that after intranasal or subcutaneous vaccine injection, chimeric-loaded PLGA NPs (with or without CpG) significantly increased the titers of IgG1, IgG2a, and sIgA antibodies, with an increased IgG2a/IgG1 ratio indicating a shift in Th1/Th2 responses toward Th1 immunity. Additionally, PLGA NPs effectively increased IFN-γ and IL-10 levels while reducing IL-4 and TGF-β1 levels, efficiently triggering Th1 immunity and inhibiting Th2 responses. PLGA NPs also improved the stability and immunogenicity of the chimera, and intranasal or subcutaneous injection could effectively induce cellular and mucosal immune responses, especially the production of mucosal sIgA antibodies, which is of great significance for the development of HTLV-1 vaccines.

Khademi et al. [69] co-encapsulated the *Mycobacterium tuberculosis* HspX/EsxS fusion antigen and monophosphoryl lipid A (MPLA) adjuvant into PLGA:DDA hybrid nanoparticles and observed the effects on mucosal and systemic immune responses in mice via intranasal administration. PLGA played an important role in the stability of the formulation and the protection of antigens. Mice intranasally inoculated with PLGA:DDA/HspX/EsxS/MPLA showed a significant increase in IFN-γ compared with other groups, and IL-4 and IL-17 levels were also significantly higher than those in other groups. The level of IgA secretion in mouse nasal washes also increased significantly. Therefore, it was inferred that this formulation could effectively induce mucosal and systemic immune responses against the HspX/EsxS antigen.

PLGA is also often modified with natural polymers. Zare et al. [70] prepared chitosan- or trimethyl CS (TMC)-coated PLGA nanospheres as a novel anti-tuberculosis vaccine. Experiments showed that intranasal vaccination significantly stimulated sIgA secretion and induced stronger mucosal immune responses, among which BCG (Bacillus Calmette–Guérin) + TMC-PLGA nanospheres induced the highest sIgA levels. Cytokine detection showed that TMC-coated PLGA nanospheres were more effective in promoting IFN-γ secretion and inducing Th1 immune responses. All vaccine formulations promoted the shift in Th1 and Th17 balance toward Th1-dominant immunity and inhibited TGF-β secretion.

### 5.2. Natural Biodegradable Polysaccharide-Based Nanoparticles

#### 5.2.1. Chitosan (CS) and Its Derivative Carriers

CS is derived from marine biological shells and exhibits non-toxicity and excellent biocompatibility. Under physiological conditions, CS nanoparticles (CS-NPs) carry positive charges, while mucins on the respiratory mucosal surface are negatively charged. Through electrostatic interaction between the two, CS-NPs easily adhere to the respiratory mucosal surface. This adhesion significantly slows down the MCC rate of CS-NPs, thereby prolonging the residence time of the vaccine on the respiratory mucosa, increasing the contact time with the mucosa, and facilitating the vaccine’s efficacy. CS can also open the tight junctions of nasal mucosal epithelial cells, enhance the permeability of the respiratory mucosa, and allow the vaccine to easily enter mucosal tissues, thereby improving the affinity between the vaccine and the respiratory mucosa and helping the vaccine better interact with mucosal immune cells to trigger immune responses [71,72,73].

For pneumococcal vaccines, the Robla team developed a CS nanocapsule (CS NC) delivery platform, enhancing antigen loading capacity and mucosal penetration through a core–shell structure design: the nanocapsules feature a hydrophobic oily core (e.g., Miglyol^®^) stabilized by a non-ionic surfactant (PEG-st 40) to prevent aggregation, along with sodium glycocholate (SGC) as a co-surfactant to provide negative charge and improve stability. CS molecules are anchored on the core surface via ionic interactions to form a hydrophilic shell. By chemically conjugating the C (PsaA) antigen with CS-maleimide-modified nanocapsules, the system demonstrated high antigen loading efficiency, excellent mucosal adhesion, favorable physicochemical stability, and strong interactions with immune cells, serving as a promising candidate for pneumococcal intranasal vaccines [74].

Xu et al. cloned the PsaA antigen gene into the eukaryotic expression vector pVAX1 via genetic engineering to construct the pVAX1-PsaA plasmid, which was then complexed with CS to prepare CS-DNA nanoparticles. This carrier achieved a DNA binding efficiency of 97.5% and could effectively protect DNA from enzymatic hydrolysis. Animal immunization experiments showed that the CS-PsaA group significantly increased the serum levels of anti-PsaA-specific IgG, IgG1, and IgG2a antibodies, enhanced IgA secretion at mucosal sites such as the nasal cavity, induced splenocytes to secrete IL-17A and IFN-γ, and ultimately reduced Streptococcus pneumoniae colonization in the nasopharynx, thus realizing the complete efficacy of “antigen delivery-immune activation-anti-infection protection” [75].

Targeting COVID-19 mRNA intranasal vaccines, the ltay Benetti team developed a lipid–CS composite nanoformulation, optimizing delivery efficiency by adjusting component ratios and environmental parameters (pH value). The optimal formulation (08LP28) consists of DOTAP/cholesterol liposomes (ratio 80:20), 85 nM PEG 2000, and 0.15 nM CS at pH 7.4. With the synergistic effect of PEG 2000 (optimizing liposome stability) and CS (enhancing mucosal uptake), the particle size of the formulation after mRNA loading was reduced to approximately 587.7 nm, and the zeta potential was maintained at around +16.5 mV, exhibiting good physical stability and biocompatibility (no cytotoxicity). Experiments confirmed that the formulation could successfully transfect mRNA in the nasal cavity, synthesize spike proteins, and induce local humoral immune responses, verifying the application potential of the intranasal delivery route for mRNA vaccines [76].

The performance of natural CS can be further optimized through chemical modification: Zhao et al. prepared nanoparticles (N-2-HACC NPs) using N-2-hydroxypropyltrimethylammonium chloride-modified CS (N-2-HACC), which showed higher stability and lower cytotoxicity than unmodified CS-NPs, and they could achieve “initial burst release-sustained stable release” of antigens. Subsequent studies showed that mice intranasally immunized with N-2-HACC-CMC/NDV NPs exhibited significantly increased serum IgG and mucosal IgA antibody titers, enhanced lymphocyte proliferation, and elevated levels of cytokines such as IL-2, IL-4, and IFN-γ, confirming that modified CS derivatives possess both vaccine carrier and adjuvant functions, making them an excellent choice for efficient mucosal vaccine delivery [77,78].

Elizabeth C. Carroll et al. published a study in Immunity in 2016, which thoroughly investigated the mechanism of action and immunomodulatory advantages of chitosan, a cationic polysaccharide, as a vaccine adjuvant. The research revealed that chitosan can specifically activate the cytoplasmic DNA sensor cGAS-STING pathway by inducing mitochondrial stress to produce reactive oxygen species (ROS) and promoting the release of endogenous DNA, thereby driving dendritic cells (DCs) to secrete type I interferons (IFNs) and facilitating their maturation. This process relies on the synergistic effect of cGAS, STING, and the type I interferon receptor (IFNAR), ultimately efficiently inducing antigen-specific Th1-type cellular immunity and IgG2c antibody responses without triggering excessive proinflammatory cytokine secretion, showing excellent safety. Additionally, intranasal immunization experiments in mice confirmed that this immune-enhancing effect of chitosan is also stably present in mucosal delivery scenarios. With its advantages of biocompatibility, mucoadhesion, and pathway-specific activation, chitosan provides an important theoretical basis for the development of mucosal vaccine adjuvants and clarifies the key mechanism by which cationic polymers can bridge innate and adaptive immunity by regulating the cGAS-STING pathway [79].

Building on this, Zhao et al. further expanded the application potential of chitosan-based adjuvants by developing chitosan derivative-based carrier nanoparticles, such as N-2-hydroxypropyl trimethyl ammonium chloride chitosan/N,O-carboxymethyl chitosan composite NPs (N-2-HACC/CMCS NPs). These NPs uniquely combine delivery functionality with intrinsic immunostimulatory activity, and their mechanism of enhancing immune responses by activating the cGAS-STING pathway is fully supported by experimental evidence. Specifically, the nanoparticles are efficiently internalized by RAW264.7 macrophages, with an uptake rate approaching completeness within 24 h, and they significantly promote the maturation of bone marrow-derived dendritic cells (BMDCs)—the proportion of CD11c^+^ positive cells reached 20.8%, which is notably higher than the 8.91% in the LPS control group. Results from qRT-PCR and Western blot analyses demonstrate that nanoparticle treatment remarkably upregulates the gene and protein expression levels of cGAS, TBK1, IRF3, and STING in macrophages (cGAS gene expression is increased by 2.2-fold and IRF3 by 7.1-fold at 24 h), thereby dose-dependently inducing the secretion of proinflammatory cytokines (IL-6, TNF-α, and IL-12p40) and type I interferons (IFN-α/β). Crucially, blocking the pathway with the cGAS inhibitor RU.521 or the STING inhibitor H-151 significantly reduces the secretion of these cytokines and interferons, directly confirming that the NPs’ immunostimulatory effect is strictly dependent on the cGAS-STING pathway [80].

This figure compares the core differences in the mucosal delivery processes of three polymeric nanoparticles (PCL, PEG-PLGA, and chitosan) as intranasal vaccine adjuvant carriers.

Mucosal penetration modes: PCL nanoparticles: Due to low adhesion to mucin, they penetrate the mucosa gradually via slow diffusion. PEG-PLGA nanoparticles: Leveraging PEG-modified properties, they cross the mucosal layer rapidly via passive diffusion. Chitosan nanoparticles: They rely on active binding between positively charged surface amino groups and mucin and penetrate the mucosa via tight junction pathways.

Antigen release characteristics: PCL nanoparticles: After endocytosis by epithelial cells, they release antigens sustainably via slow hydrolytic degradation. PEG-PLGA nanoparticles: Post-internalization, they achieve rapid antigen release via fast hydrolytic degradation. Chitosan nanoparticles: After mucosal penetration, they degrade and release antigens gradually via enzymatic degradation.

#### 5.2.2. Alginate and Its Composite Carriers

While natural cationic polysaccharides like CS excel in mucosal adhesion and barrier penetration, natural anionic polysaccharides have gained attention for their unique structural advantages in antigen binding and formulation stability—with alginate being a representative example.

Alginate is a natural polysaccharide extracted from brown algae and is mainly composed of two uronic acid monomers, β-D-mannuronic acid (M) and α-L-guluronic acid (G), which are linearly linked via 1,4-glycosidic bonds to form high-molecular-weight polymers. The distribution of the M and G units in the molecular chain can be continuous, alternating, or random. Its core structural feature lies in the large number of carboxyl groups on the molecular chain—these carboxyl groups can ionize under different pH conditions, endowing alginate with negative charges. This characteristic not only grants it the ability to undergo electrostatic interactions with positively charged substances but also lays a structural foundation for its binding to antigens and drug molecules, as well as interactions with cell membrane surfaces when used as a vaccine adjuvant or delivery system [81].

Based on this structural advantage, alginate exhibits significant efficacy in vaccine applications: on the one hand, it can protect antigens from damage caused by the physiological environment by forming specific structures, achieving sustained antigen release, prolonging the in vivo action time of antigens, and thereby enhancing immune stimulation effects; on the other hand, when used in combination with substances such as CS and CpG ODN, it can produce synergistic effects to further improve immune responses. As a vaccine adjuvant or delivery system, the core efficacy advantages of alginate are reflected in three dimensions: first, improving vaccine immunogenicity and enhancing the body’s resistance to viruses; second, possessing excellent biocompatibility and safety, reducing the risk of adverse effects; and third, holding the potential to reduce vaccine dosage and costs, providing strong support for the development of safer and more effective vaccines [82].

In specific application studies, the correlation between structural design and efficacy performance is particularly prominent. Dehghan et al. prepared alginate nanoparticles via ionic gelation technology, incorporating an influenza vaccine and CpG-ODN into the nanoparticles. In terms of structural protection efficacy, the encapsulation process did not affect the structure and chemical properties of proteins—SDS-PAGE analysis showed no obvious aggregation or degradation of influenza virus proteins during nanoparticle synthesis, confirming that alginate nanoparticles can effectively protect the structural integrity of antigens and ensuring their immunogenicity remains intact. Regarding biosafety, XTT cytotoxicity assay results indicated that both blank alginate nanoparticles and those loaded with antigens/adjuvants had no significant impact on the growth and viability of Calu-6 cells, with high cell survival rates, fully verifying their good biocompatibility. In terms of immune efficacy, after intranasal administration, the combination of CpG-ODN and the influenza virus induced strong humoral and cellular immune responses, further highlighting the role of alginate carriers in synergistically enhancing immune effects [83].

Composite biomaterials formed by coating CS with alginate through electrostatic interactions have achieved synergistic optimization of structure and efficacy—this composite carrier combines the cationic properties of CS and the anionic properties of alginate, forming a unique core–shell structure. It not only exhibits significantly improved stability but also allows precise regulation of the particle size, surface charge, and morphology of composite particles by adjusting the ratio of the two components and coating conditions, thus expanding its application potential in vaccine delivery. In a study targeting *Mycobacterium tuberculosis*, researchers co-administered alginate-coated CS nanoparticles loaded with the *Mycobacterium tuberculosis* proline–proline–glutamic acid (PPE) protein (PPE17) with the ISCOMATRIX nanoadjuvant via the intranasal route. ISCOM-like nanoparticles can break through the mucosal barrier by promoting the uptake of antigens by mucosal DCs and activating related immune cells and pathways, while alginate-coated CS nanoparticles achieve sustained antigen release (in vitro release lasting 144 h) through their core–shell structure. This structural design prolongs the contact time between the vaccine and the respiratory mucosa, enhances affinity, and improves the efficiency of antigen penetration through the epithelial layer; under the synergistic effect of the two nanoparticle types, the production of specific anti-PPE17 serum antibodies (IgG1 and IgG2a) and sIgA in mice was significantly increased, effectively enhancing vaccine immunogenicity [84].

Particle size regulation, as an important direction of structural optimization, has also brought significant efficacy improvements. Mosafer et al. prepared alginate-coated CS and trimethyl chitosan (TMC) nanoparticles loaded with the PR8 influenza virus. The structural modification of the alginate coating increased the particle size and reduced the zeta potential of the nanoparticles while endowing them with good stability. Immunization experiments showed that both PR8-CS and PR8-TMC nanoparticles significantly improved immune protection and induced mixed Th1/Th2 immune responses. Among them, alginate-coated PR8-TMC nanoparticles (PR8-TMC-ALG) exhibited superior Th1 immune response induction efficacy—they produced higher IgG2a antibody titers, and their IgG2a/IgG1 ratio was significantly higher than that of other groups. This confirms that structural modifications (coating and particle size regulation) can precisely optimize the type of immune response and enhance the targeted protective effect of vaccines [85].

Notably, the enhanced Th1-biased immune response induced by such particle size-regulated and coated chitosan-based nanoparticles is closely associated with the potential activation of the cGAS-STING pathway. Previous studies have demonstrated that chitosan and its derivatives (such as TMC) can trigger mitochondrial stress in immune cells after intracellular delivery, leading to the release of mitochondrial DNA (mtDNA) into the cytoplasm. The appropriately sized alginate-coated nanoparticles facilitate efficient endocytosis by antigen-presenting cells (APCs) such as dendritic cells (DCs) and macrophages, and the subsequent release of encapsulated viral antigens and chitosan derivatives promotes the exposure of mtDNA to cytosolic cGAS. This activates the cGAS-STING pathway, triggering the secretion of type I interferons (IFNs) and proinflammatory cytokines (e.g., TNF-α and IL-12), which in turn amplify the differentiation of Th1 cells and the production of IgG2a antibodies—consistent with the superior Th1 immune response observed in PR8-TMC-ALG nanoparticles. The synergistic effect of particle size regulation (improving cellular uptake efficiency) and coating modification (enhancing stability and mucosal adhesion) not only optimizes the delivery of antigens and adjuvants but also creates favorable conditions for the activation of the cGAS-STING pathway. This further verifies that the rational structural design of nanoparticles can synergize with the cGAS-STING pathway to precisely regulate immune response types, providing a valuable technical reference for the development of high-efficacy influenza mucosal vaccines [86].

#### 5.2.3. α-Glucan Nanoparticles

α-Glucan is a naturally occurring polysaccharide composed of glucose monomers linked by α-glycosidic bonds, and it is widely sourced from plants (e.g., corn phytoglycogen) and microorganisms. When fabricated into nanoparticles, it exhibits enhanced mucosal penetration and targeted immune cell recognition, making it a promising intranasal adjuvant.

Patil et al. fabricated cationic amphiphilic nanoparticles (Nano-11) via chemical modification using corn-derived α-D-glucan (phytoglycogen) as the raw material. As an intranasal vaccine adjuvant, this nanoparticle can activate immune responses by directly or indirectly binding to receptors such as NLRP3 and Dectin-1, induce balanced Th1/Th2 cytokine secretion and follicular helper T-cell (Tfh) activation, and elicit strong cross-protective immunity after intranasal immunization. Its mechanism of action involves NLRP3 inflammasome activation, electrostatic membrane perturbation, and epigenetic regulation-mediated trained immunity. Specifically, NLRP3 inflammasome activation is triggered when Nano-11 binds to receptors on the surface of immune cells, leading to intracellular events including reactive oxygen species (ROS) generation and potassium ion efflux. These signals promote the assembly of NLRP3, apoptosis-associated speck-like protein containing a CARD (ASC), and caspase-1 into a functional complex, which cleaves pro-IL-1β and pro-IL-18 into their mature forms for extracellular secretion, thereby enhancing local mucosal inflammatory responses and immune cell recruitment [87].

In the study by Weidong Zhang et al., α-glucan (dextran, a member of the α-glucan family) was used as the core carrier, which precisely addressed the key challenge that the nucleic acid adjuvant CpG has difficulty targeting lymph nodes, resulting in limited antitumor efficacy. In this research, α-glucan was conjugated with CpG through chemical bonding. It not only ensured the safety of the carrier by virtue of its excellent biocompatibility but also significantly increased the hydrodynamic size of CpG via a size-regulating effect—confining it within the ideal lymphatic targeting range of 5–200 nm. This avoided the drawback of free CpG being rapidly cleared through the bloodstream, enabling efficient targeted enrichment of the adjuvant in lymph nodes and substantially improving the accumulation efficiency of CpG at the initiation site of lymph node immune responses. Meanwhile, the conjugated structure of α-glucan optimized the delivery stability of CpG and enhanced its targeted binding ability to immune cells such as dendritic cells in lymph nodes. Ultimately, it significantly strengthened antigen presentation and CD8^+^ T-cell activation, remarkably improving the efficacy of antitumor immunotherapy. The study provides crucial technical support and a theoretical basis for the application of α-glucan-mediated targeted adjuvant delivery in antitumor immunotherapy [88].

#### 5.2.4. β-Glucan Nanoparticles

β-Glucan is a polysaccharide with a backbone of β-(1,3)-linked glucose residues (often with β-(1,6) side branches) and is primarily derived from fungi, yeast, and mushrooms. It is well-known for its immunostimulatory activity via binding to pattern recognition receptors (PRRs) like Dectin-1 and TLR2/6, and nanoformulation further optimizes its mucosal delivery efficiency.

Yang et al. prepared fungal β-glucan self-assembled nanoparticles by nanoprecipitation. When the degree of hydrophobic substitution (DS) was optimized and the organic phase/water phase ratio was 10:1, the nanoparticles were the smallest (approximately 95 nm) with optimal nasal mucosal penetration and stability. As an intranasal influenza vaccine carrier, this nanoparticle can encapsulate antigens, prolong mucosal retention time, enhance mucosal sIgA and systemic IgG antibody responses by activating macrophages and dendritic cells (DCs), and exhibit excellent biocompatibility without obvious mucosal toxicity, providing key preparation technologies and immune data for the development of polysaccharide carriers for intranasal vaccines [89].

Hilliard L. Kutscher et al. utilized β-glucan as a core immunostimulatory and targeting ligand to construct β-glucan–chitosan (CS)–poly(lactic-co-glycolic acid) (PLGA) composite nanoparticles (β-C-P) for the inhalational delivery of rifampin (Rif) in the treatment of tuberculosis (TB). With PLGA as the core to load rifampin and chitosan as the intermediate layer, the nanoparticles are surface-modified with β-glucan. By specifically binding to the Dectin-1 receptor on the surface of alveolar macrophages, β-glucan not only enhances the cellular targeting and phagocytic efficiency of the nanoparticles but also activates the innate immune response—significantly inducing the secretion of proinflammatory cytokines such as TNFα, IL-6, and IL-1β and promoting the recruitment and activation of immune cells including alveolar macrophages, T cells, and neutrophils—without causing damage to the alveolar epithelium. Pharmacokinetic studies showed that after oropharyngeal aspiration (OPA) administration, the nanoparticles can sustainably release rifampin in the lungs of mice for up to 7 days, with significantly higher drug exposure in bronchoalveolar lavage fluid (BAL) compared to free rifampin, while reducing systemic drug exposure to minimize side effects. The incorporation of β-glucan is crucial for the formulation to achieve the dual functions of “targeted drug delivery + immune stimulation,” laying a foundation for the development of a weekly inhalational adjuvant immunotherapy regimen for TB and effectively addressing the problems of poor drug permeability, a long treatment course, and low patient compliance in traditional TB treatment [90].

### 5.3. Lipid Nanoparticles

Natural polysaccharide-based carriers, through single modifications or composite designs, have demonstrated synergistic advantages in antigen delivery and immune enhancement. However, there is still room for improvement in balancing the simplicity of large-scale production and biocompatibility. In contrast, lipid nanoparticles, with their unique membrane structure and biocompatibility, have emerged as another core research direction in the field of intranasal vaccine carriers.

Liposomal nanoparticles are closed vesicular structures composed of a bilayer membrane made from lipid components such as phospholipids. Compared with polymeric nanoparticles, they exhibit superior safety and degradability and are easier to prepare. Liposomal nanoparticle carriers for nasal administration are typically fabricated using cationic lipids. Owing to their positively charged surface, these carriers facilitate interactions with the negatively charged surface of nasal mucosal epithelial cells or tight junction regions when passing through the nasal mucosal barrier. This interaction affects the tight junctions and promotes the carriers to cross the nasal mucosal barrier via the paracellular pathway [91,92]. However, core drawbacks of intranasal lipid nanoparticles include excessive stimulation and inflammation induced by cationic lipids: their surface positive charges strongly interact with negatively charged nasal mucosal epithelial cells and mucus, disrupting mucus integrity and epithelial membrane stability, causing hyperemia, edema, inflammatory infiltration, and even epithelial cell apoptosis/necrosis. Excessive cationic lipids also non-specifically activate immune cells, triggering inflammatory cascades with abnormal secretion of pro-inflammatory cytokines (e.g., IL-6 and TNF-α), exacerbating local damage and impairing mucociliary clearance function. This toxicity reduces vaccination tolerability, impairs mucosal barrier function to affect antigen delivery, and becomes a key bottleneck for clinical translation, requiring targeted improvements via lipid combination optimization, surface charge regulation, and functional component modification.

Studies have shown that cationic dimethyldioctadecylammonium (DDA)/trehalose 6,6′-dibehenate (TDB) liposomes, when used as mucosal vaccine adjuvants and administered intranasally, can effectively induce both mucosal and systemic immune responses, making them a promising vaccine delivery carrier. The positive charge of DDA can prolong the retention time of antigens at the injection site, forming a depot effect that increases antigen presentation and promotes the activation and maturation of immune cells, thereby enhancing the immune response. Additionally, DDA can efficiently bind to anionic antigens to form a stable system. When combined with TDB (trehalose 6,6′-dibehenate—a synthetic immunostimulant often used with cationic lipids such as DDA to form cationic liposomes) and with H3N2 selected as the antigen, experimental results demonstrated that mice intranasally immunized with DDA/TDB liposomes encapsulating the H3N2 antigen exhibited a significantly higher H3N2-specific sIgA antibody response in nasal lavage fluid compared to other groups. The levels of H3N2-specific immunoglobulin G (IgG), IgG1, and IgG2b antibodies in serum also increased significantly. In vitro experiments further indicated that these liposomes significantly promoted dendritic cell (DC) maturation, as evidenced by notably elevated levels of the CD80, major histocompatibility complex class II (MHC-II), and CD86 markers. Meanwhile, regarding the potential toxicity issues associated with DDA cationic lipids, the strategy of combining DDA with TDB has also achieved a favorable improvement in mitigating toxicity. As a glycolipid immunomodulator, TDB not only synergizes with DDA to enhance immune responses but also regulates the fluidity of the liposomal membrane and the distribution of surface charges. This reduces the risk of local mucosal irritation caused by DDA alone, decreases inflammatory cell infiltration, and simultaneously leverages the synergistic effect between the two components to lower the dosage of each individual component while ensuring immune efficacy, thereby indirectly reducing toxin accumulation [93].

While DDA/TDB liposomes have demonstrated robust mucosal and systemic immune activation, their clinical applicability is partially constrained by suboptimal cellular uptake efficiency and potential rapid clearance by the immune system. Recent studies have also constructed cationic liposomal influenza vaccines (DDA-DSPC-TPGS) modified with d-α-tocopheryl PEG 1000 succinate (TPGS). When administered via the nasal mucosa, these vaccines effectively enhance immune responses, providing a new direction for influenza vaccine development. TPGS is a polar head group PEG derivative of natural vitamin E succinate. Acting as an edge activator and immunoadjuvant in nanosystems, it can insert into the hydrophobic layer of DDA-DSPC liposomes. The PEG structure of TPGS extends the circulation time of the nanodrug delivery system and significantly improves the efficiency of cellular uptake of the vaccine, thereby enhancing the immune response. Furthermore, the authors found that compared to the cationic liposome control group, TPGS-modified liposomes reduced the zeta potential of the liposomes, altered their surface charge, and decreased the likelihood of rapid phagocytosis of the liposomes. They also significantly reduced cytotoxicity toward bone marrow-derived dendritic cells (BMDCs) and DC2.4 cells. Furthermore, this study adopted TPGS modification as the core strategy to address the toxicity issue of lipid nanoparticles: on the one hand, the PEG segment of TPGS can form a hydrophilic protective layer on the liposome surface, weakening the non-specific electrostatic interaction between cationic lipids and cells and reducing cell membrane damage and apoptosis; on the other hand, as a natural vitamin E derivative, TPGS itself possesses excellent biocompatibility and antioxidant activity, allowing it to neutralize the accumulation of reactive oxygen species (ROS) induced by cationic lipids to reduce cytotoxicity. Meanwhile, its edge activator property can optimize the liposome structure, decrease the release of free cationic lipids, and further mitigate the risk of mucosal irritation [86].

Beyond the optimization of DDA-based liposomes, researchers have explored other cationic lipid combinations to expand the applicability of lipid nanoparticle carriers for intranasal vaccines. A study developed an intranasal vaccine based on DOTAP cationic liposomes and pneumococcal surface protein A (PspA) using a combination of DOTAP/DC-chol (cholesterol-3β-N-(dimethylaminoethyl) carbamate) as the carrier. DC-chol is a cationic lipid widely used in vaccine research; it is often combined with DOTAP to prepare cationic liposomes and plays a key role in improving the immune efficacy of vaccines. Experiments revealed that DOTAP/DC-chol induced a more comprehensive immune response and significantly reduced mortality compared to DOTAP alone. Intranasal immunization with PspA combined with DOTAP/DC-chol liposomes enhanced antigen uptake by inducing PspA-specific antibodies and T helper 17 (Th17) immune responses, thereby conferring protection against Streptococcus pneumoniae infection in mice. Thus, this cationic liposome represents a promising mucosal adjuvant [94].

DOTAP is widely used in mRNA vaccines. A study selected a positively charged protamine–mRNA vaccine that encapsulated the mRNA to form stable cationic–mRNA complexes. These complexes were further encapsulated and protected using DOTAP/cholesterol/distearoylphosphatidylethanolamine (DSPE)–PEG cationic liposomes (LPC). Research findings showed that the dual protection of protamine and cationic liposomes significantly improved the stability of the vaccine. Additionally, LPC/mRNA effectively promoted antigen uptake, DC maturation, and cytokine secretion, thereby inducing cellular immune responses. LPC/mCK19 (LPC encapsulating mRNA encoding mouse cytokeratin 19) demonstrated excellent antitumor efficacy in a Lewis lung cancer model and is expected to be a candidate vaccine for intranasal immunotherapy, though further validation in other tumor models is required [95].

CAF 01 is a novel liposomal adjuvant system comprising a structurally stable cationic liposomal carrier (DDA) and a glycolipid immunomodulator (trehalose 6,6′-dibehenate (TDB)). Studies have indicated that intranasal vaccination with CAF 01-based vaccines for the prevention of influenza or Streptococcus pyogenes effectively induces the production of mucosal effector T cells and immunoglobulin A (IgA) immune responses while also providing protection to immunized model animals. Furthermore, the level of antigen-specific interferon-gamma (IFN-γ) response produced by the spleens of CAF 01-immunized mice was four times higher than that of mice immunized with the non-adjuvanted vaccine. This finding suggests that CAF 01 significantly enhances the level of vaccine-specific serum IgG. Meanwhile, after structural optimization, CAF 01 forms a more stable liposomal structure through the combination of DDA and TDB, which can reduce the free release of DDA. At the same time, TDB can precisely regulate immune signals to avoid nonspecific inflammation, achieving more thorough toxicity control and higher biosafety. Its core advantage lies in potently activating cellular immunity, thereby significantly enhancing antigen-specific IFN-γ secretion and mucosal effector T-cell responses. It is compatible with various antigen types such as viruses and bacteria and is particularly suitable for the development of vaccines requiring long-term protection against intracellular pathogens. Additionally, it exhibits strong structural stability and a more favorable shelf life [96].

### 5.4. Saponin-Based Composite Carrier Adjuvants Such as ISCOMATRIX

ISCOMATRIX is a cage-like nanoadjuvant self-assembled from saponin, cholesterol and phosphatidylcholine via the lipid film hydration method, with a particle size of approximately 82.4 nm and a negative surface charge of −18.6 mV. It contains no preloaded antigens and can load antigens such as *Mycobacterium tuberculosis* HspX/EsxS through adsorption or conjugation.

Against the backdrop of the limited protective efficacy of the BCG vaccine against tuberculosis, a study investigated the mucosal and systemic immunomodulatory effects of the ISCOMATRIX nanoadjuvant combined with alginate-coated chitosan nanoparticles loaded with the *Mycobacterium tuberculosis* PPE17 antigen via intranasal and subcutaneous administration in mice. First, cage-like ISCOMATRIX adjuvants with a particle size of 59 ± 6 nm and a zeta potential of −9.1 ± 0.4 mV were prepared by the lipid hydration method. Sixty BALB/c mice were divided into 10 groups (four control groups and six immunization groups), and after three immunizations on days 0, 14 and 28, the levels of serum antibodies, secretory IgA (sIgA) in nasal lavage fluid and cytokines in splenocytes were detected. The results showed that the combination of ISCOMATRIX and nanoparticles via both administration routes could significantly induce mice to produce high levels of Th1-type cytokines (IFN-γ and IL-17) as well as serum IgG2a and IgG1 antibodies; intranasal administration also efficiently elicited mucosal sIgA secretion, with the IFN-γ level induced by intranasal administration being significantly higher than that by subcutaneous administration, while subcutaneous administration exhibited better efficacy in inducing IL-17 and IL-4 and acted as a BCG booster. This study confirmed that ISCOMATRIX is a highly promising vaccine adjuvant that can enhance the cellular and humoral immune responses to tuberculosis antigens via intranasal and subcutaneous routes and that the immunization route and prime-boost strategy can affect the type of Th1/Th2 immune responses it induces, thus providing important experimental evidence for the development of novel tuberculosis vaccines [84].

A study evaluated the immunogenicity of the *Mycobacterium tuberculosis* HspX/EsxS fusion protein combined with the ISCOMATRIX–PLUSCOM nanoadjuvants and MPLA via intranasal administration, and its core immune-enhancing mechanism is closely associated with ISCOMATRIX-mediated NLRP3 inflammasome activation. As a cage-like nanoadjuvant self-assembled from saponin, cholesterol and phosphatidylcholine (with a particle size of approximately 82.4 nm and a surface charge of −18.6 mV), ISCOMATRIX can be efficiently internalized by mucosal antigen-presenting cells (APCs) after intranasal delivery, and it promotes the secretion of the proinflammatory cytokine IFN-γ by activating the NLRP3 inflammasome pathway while exerting a synergistic effect with MPLA to further boost the immune response. Experimental results showed that both the HspX/EsxS/ISCOMATRIX/MPLA formulation and its BCG booster group could significantly induce higher levels of serum IgG1 and IgG2a antibodies as well as secretory IgA (sIgA) in nasal lavage fluid, with IFN-γ levels markedly higher than those in the HspX/EsxS antigen-only group and the BCG group. These findings confirm that ISCOMATRIX enhances the mucosal and systemic immunogenicity of tuberculosis antigens by activating the NLRP3 inflammasome, thus providing experimental evidence for the development of novel intranasal tuberculosis vaccines or BCG boosters [97].

### 5.5. Inorganic Nanoparticle Carriers

Cationic liposomes and innovative adjuvant systems such as CAF 01 have anchored lipid nanoparticles as a robust, well-established pillar for intranasal vaccine delivery by leveraging their exceptional biocompatibility and potent immune-activating capacity. Yet, moving beyond the conventional paradigm of polymeric and lipid-based carriers, inorganic nanoparticle delivery systems are carving out a unique niche in this field—their unparalleled physicochemical properties addressing long-standing unmet needs in vaccine stability and targeted delivery, thus establishing them as a highly differentiated research frontier.

Compared with other nanoparticle carriers, inorganic nanoparticle carriers have advantages such as high stability—they can maintain structural and functional stability in complex physiological environments. They also possess good biocompatibility, rarely triggering severe immune responses, and can be administered through multiple routes, expanding their application scenarios. However, challenges remain in terms of cost, manufacturing processes, and potential toxicity, which need to be addressed.

Gold nanoparticles (AuNPs) are used as vaccine carriers due to their excellent biocompatibility and modifiability. In the early stage, there were many applications of AuNPs in influenza vaccines [98,99,100]. Recently, Ingrole et al. [101] developed an M2e (highly conserved matrix protein 2 of influenza A virus)-conjugated AuNP influenza vaccine with both thermal stability and immunological efficacy. The authors immunized mice with the reconstituted vaccine under different storage conditions. Compared with the newly prepared vaccine group, the immunized mice all produced significant M2e-specific IgG antibody responses, and there was no significant difference in IgG1 and IgG2a antibody responses, indicating that storage conditions did not affect the immunogenicity of the vaccine. In the ferret immunization experiment, intramuscular injection and nasal administration were compared. It was found that 63 days after immunization, the M2e-specific IgG antibody level in the nasal administration group was superior. After intranasal virus inoculation, the virus titer in the nasal administration group was significantly reduced, while there was no significant change in the intramuscular injection group.

The potential of gold nanoparticles (AuNPs) in enhancing vaccine stability and mucosal immunogenicity has been fully validated, while organic–inorganic hybrid nanoparticles (such as polyphosphazene-based nanoparticles) have provided a novel direction for the development of intranasal multivalent vaccines by virtue of their multi-antigen loading capacity and synergistic immunomodulatory properties.

Polyphosphazenes are a new class of organic–inorganic hybrid polymers with a main chain composed of alternating nitrogen and phosphorus atoms, featuring excellent biocompatibility and non-toxic degradability. Furthermore, organic–inorganic hybrid nanoparticles can load multiple antigens or adjuvants simultaneously, enabling the delivery of multivalent vaccines.

Aibani et al. [102] prepared an acellular pertussis vaccine combined with a three-adjuvant system. They combined pertussis toxin, pertussis toxin, and fimbriae 2/3 antigens with the innate defense regulatory peptide IDR 1002, the TLR 3 agonist Poly I:C, and polyphosphazene. In the experiment, two types of antigens (L-TriAdj external-associated antigen (LT-A) and L-TriAdj internal-associated antigen (LAT)) were mixed with IDR 1002, Poly I:C, and polyphosphazene: the former (LT-A) was combined through electrostatic attraction, while the latter (LAT) was achieved by the antigen residing inside lipid nanoparticles. Experimental results demonstrated that compared with commercial acellular pertussis vaccines, intranasal administration of this formulation produced a more compact and uniform particle system, significantly increasing the content of sIgA in the nasal cavity as well as the serum antibody titers of immunoglobulin G2a (IgG2a) and immunoglobulin A (IgA). Additionally, it stimulated a more balanced T helper 1 (Th1)/T helper 2 (Th2)-type systemic and mucosal immune response, and compared with LAT, LT-A nanoparticles tended to induce higher serum antibody titers.

Other studies have revealed that mesoporous silica nanoparticles (MSNs)—a common basic material for silica–polymer hybrid nanoparticles—possess a high specific surface area and adjustable pore sizes, allowing them to efficiently load a variety of therapeutic agents, including small molecules, genes, peptides, and proteins. To improve the performance of MSNs, organic polymers are often introduced for surface modification: PEG can enhance the hydrophilicity of nanoparticles, reduce protein adsorption, prolong in vivo circulation time, and decrease the clearance rate by the immune system; polyethyleneimine (PEI) can improve the transfection efficiency of nanoparticles, facilitating gene delivery; and CS has good biocompatibility and biodegradability, allowing it to enhance the mucosal adhesion of nanoparticles. Moreover, the large number of silanol groups on the surface of MSNs facilitates functional modification, and targeted delivery to specific tissues or cells can be achieved after conjugation with targeting ligands. It can be inferred that for intranasal vaccines, MSNs can enable the vaccine to act more precisely on nasal-related immune cells, thereby enhancing the immune response—for example, modified MSNs can specifically bind to receptors on the surface of immune cells in the nasal cavity, promoting the uptake of the vaccine by immune cells and triggering a stronger immune response. Although no literature has mentioned the application of MSNs in intranasal vaccines, there are numerous successful studies in other mucosal administration routes (such as oral and intestinal routes) and disease treatment fields; in tumor and diabetes treatment, for instance, MSNs can load drugs to achieve the synergy of multiple therapeutic approaches. These research findings provide strong references for the application of MSNs in the field of intranasal vaccines, indicating their potential application value in intranasal vaccines [103,104,105]. Whether it is synthetic polymers, natural polysaccharides, or liposomal carriers, their immune-enhancing potential has been verified through laboratory optimization. However, transitioning from being effective in the laboratory to being clinically applicable requires overcoming multiple hurdles, including large-scale production, quality control, and regulatory approval. These practical challenges collectively constitute the final stretch in the clinical translation of intranasal vaccine.

### 5.6. Ligand-Mediated Targeted Delivery in Adjuvant–Carrier Systems

Targeted delivery strategies for nasal mucosal immunity are centered on “receptor-ligand specific binding.” By precisely recognizing characteristic receptors on the surface of nasal mucosal immune cells (e.g., antigen-presenting cells) or epithelial cells, these strategies achieve the directional enrichment and efficient uptake of vaccines/antigens. They address the core limitations of traditional intranasal vaccines, such as short mucosal retention, poor immune cell targeting, and significant off-target effects, while enhancing immune efficacy and reducing systemic toxicity. This provides critical technical support for the clinical translation of low-immunogenicity formulations like subunit vaccines.

#### 5.6.1. FcRn-Mediated Albumin-Targeted Delivery

The neonatal Fc receptor (FcRn) is highly expressed in nasal mucosal epithelial cells, with a natural function of mediating the transmucosal transport and homeostasis of IgG and albumin. This property has been widely exploited to optimize the targeted delivery of intranasal vaccines. Covalent conjugation of vaccine antigens or carrier nanoparticles with human serum albumin (HSA) enables efficient transmucosal delivery of antigens via the “pH-dependent binding-transport” mechanism of FcRn. In the neutral nasal mucosal environment, albumin binds to FcRn with low affinity and is endocytosed by epithelial cells into endosomes (pH 5.0–6.0). Under acidic conditions, the affinity between the two is significantly enhanced, preventing antigen degradation by lysosomes. Finally, antigens are released into the lamina propria via endosomal recycling and taken up by antigen-presenting cells (DCs and macrophages) [106]

The core advantages of this strategy have been validated by multiple studies: albumin-fused antigens increase nasal mucosal uptake efficiency by 3.2-fold, significantly prolong local mucosal retention time (from 4 h to over 12 h), and induce higher titers of secretory immunoglobulin A (sIgA) and lung-resident memory T cells (TRM), providing long-term mucosal protection against respiratory pathogen infections. Additionally, the biocompatibility of albumin reduces nasal mucosal inflammation, addressing the mucosal irritation issues of traditional delivery systems such as cationic carriers [106].

Ochsner et al. constructed an FcRn-mediated nasal mucosa-targeted influenza vaccine delivery system using trimeric HA-Fc fusion proteins—integrating influenza hemagglutinin (HA), the T4 bacteriophage fibritin foldon domain, and the Fc fragment of mouse IgG2a, with wild-type (HA-Fc/wt) and FcRn-binding defective mutant (HA-Fc/mut) variants—which showed that HA-Fc/wt specifically binds FcRn under acidic conditions while retaining HA’s trimeric conformation and antigenic activity; intranasal immunization of wild-type mice with HA-Fc/wt plus the CpG adjuvant induced high levels of serum neutralizing antibodies, respiratory mucosal IgA, and lung-resident memory T cells (TRM); activated germinal center responses; generated bone marrow long-lived plasma cells; significantly reduced lung viral loads and inflammatory damage after a lethal influenza A/PR/8/34 (PR8) challenge; and achieved an 84% survival rate with protective efficacy lasting up to 8 weeks, whereas no such effects were observed in FcRn-knockout mice or those immunized with HA-Fc/mut, confirming that FcRn-mediated nasal delivery enables efficient trimeric HA transport and robust, long-lasting local and systemic immune responses, thus providing a novel strategy for universal respiratory vaccine development [107].

#### 5.6.2. Mannose Receptor-Mediated Immune Cell-Targeted Delivery

The mannose receptor (MR, CD206), a characteristic receptor on the surface of antigen-presenting cells (APCs) such as dendritic cells (DCs) and macrophages, exhibits high specific expression, making it an ideal target for immune cell targeting of intranasal vaccines. This strategy modifies the carrier surface with mannose ligands (e.g., mannose-modified chitosan) and leverages receptor–ligand-specific binding to promote efficient endocytosis of the carrier by DCs, thereby enhancing antigen presentation efficiency.

Experimental data demonstrate that mannose-modified chitosan nanoparticles increase the uptake rate by nasal mucosal DCs by 2.8-fold compared to unmodified carriers. They can significantly induce Th1-type immune responses (with a 40% increase in IFN-γ secretion) and enhance protective efficacy against respiratory pathogens such as the influenza virus and *Mycobacterium tuberculosis*. Unlike albumin targeting, which focuses on “transmucosal transport,” mannose targeting emphasizes “precise enrichment of immune cells,” and the two can form functional complementarity: albumin efficiently delivers antigens to the lamina propria, while mannose further guides antigens to DCs, maximizing immune activation efficiency [108].

#### 5.6.3. RBD Trimer-Mediated Mucosal Epithelial-Targeted Delivery

The receptor binding domain (RBD) is a key functional region on the S1 subunit of the spike (S) protein of severe acute respiratory syndrome coronavirus 2 (SARS-CoV-2). It serves as the core structural domain for the virus to bind to host cells and achieve invasion, and it is also the major antigenic target that induces the body to produce neutralizing antibodies, making it one of the core antigen choices for the development of COVID-19 vaccines [109].

Lei et al. developed an MF59-like oil-in-water adjuvanted intranasal subunit vaccine, with the trimeric RBD of SARS-CoV-2 XBB.1.5 as the core targeting ligand. It achieves precise ACE2 receptor-mediated delivery via the specific binding of the RBD to ACE2 receptors on respiratory mucosal epithelial cells, boasting three core targeting advantages: precise anchoring to viral infection sites for improved mucosal retention; targeted immune activation at mucosal sites to induce sIgA, lung T_RM, and germinal center reactions for a mucosal immune barrier; and broad, durable protection that generates high-titer neutralizing antibodies against XBB-lineage variants (XBB.1.5 and EG.5.1) and effective neutralization of immune-evasive emerging variants (JN.1 and KP.2/KP.3), with protective antibodies persisting for at least 6 months. Moreover, this strategy serves as a potent heterologous booster for mRNA vaccines (enhancing mucosal and systemic immunity vs. homologous boosting), and the “IM + IN” combined regimen compensates for the insufficient systemic immunity of single intranasal delivery, achieving comprehensive upper- and lower-respiratory-tract protection with good safety and no obvious mucosal irritation, thus providing an efficient targeted delivery scheme for the clinical translation of COVID-19 mucosal vaccines [110].

## 6. Clinical Transformation Bottlenecks

Despite the promising advances in adjuvant design, carrier optimization, and intelligent delivery technologies summarized above, intranasal vaccines still face multiple intertwined challenges in clinical transformation. As a successful paradigm for the clinical translation of intranasal vaccines, the COVID-19 intranasal vaccine iNCOVACC (BBV154) developed by India’s Bharat Biotech holds significant reference value and provides a feasible model for addressing translation challenges. This vaccine uses PLGA nanoparticles as carriers to encapsulate the SARS-CoV-2 spike protein and is combined with a low dose of CpG (15 μg/dose) as an adjuvant. Its successful translation relies on two core strategies: on the one hand, optimizing the double emulsion preparation process by adopting a continuous-flow emulsification system, which controls the particle size coefficient of variation within 5% and increases mass production efficiency to 50,000 doses per batch [12], and on the other hand, leveraging the sustained-release property of PLGA to maintain the local concentration of CpG in the nasal mucosa at 0.5 μg/mm^2^ (below the toxicity threshold of 1 μg/mm^2^), resulting in a mucosal irritation rate of only 2.1% in Phase III clinical trials [13,111]. As a rapidly approved intranasal COVID-19 vaccine, the success of iNCOVACC confirms the unmet clinical need for “convenient administration and rapid establishment of a mucosal barrier to block respiratory virus transmission”—especially in scenarios of large-scale population immunization and emergency responses to outbreaks, where traditional injectable vaccines exhibit significant limitations in administration efficiency and inadequate mucosal protection.

Despite the breakthrough translation of iNCOVACC, intranasal vaccines as a whole still face numerous challenges in clinical translation. At the carrier and adjuvant levels, novel carriers such as polyphosphazenes and MSNs have demonstrated excellent immune-enhancing potential in laboratory studies, and their key properties have been further optimized through surface modifications. However, these positive laboratory-scale outcomes have not yet been effectively translated into clinically available vaccine products. The translation from basic research to clinical application requires comprehensive verification of the safety and efficacy of carriers, and it also needs to address a series of practical challenges such as large-scale production, quality control and cost control—among which the large-scale production of carriers and adjuvants is a key challenge. The translation of intranasal vaccine adjuvants and carriers from laboratory-scale preparation to industrial mass production constitutes a critical bottleneck: most laboratory-developed technologies struggle to maintain the consistency of key quality attributes during scale-up. For instance, when manufacturing PLGA nanoparticles for intranasal vaccines, even subtle fluctuations in critical process parameters such as emulsification speed or solvent evaporation rate during batch production may lead to obvious variations in particle size and antigen encapsulation efficiency, which may make it difficult to meet the stringent uniformity requirements for clinical-grade pharmaceutical products. Additionally, the high cost of raw materials and the complexity of post-production purification procedures for novel carriers result in significantly higher production costs for intranasal vaccines compared to traditional injectable vaccines.

Furthermore, a critical yet underemphasized barrier to the clinical translation of intranasal vaccines is the lack of standardized clinical evaluation tools and unified efficacy endpoints. A recent multicenter clinical trial of an intranasal Omicron vaccine confirmed that even healthy individuals with similar nasal mucosal spike-specific sIgA titers show markedly different respiratory virus clearance rates. Mechanistic analysis has identified two key influencing factors: first, the antigen affinity of sIgA, where high-affinity sIgA accelerates viral load reduction and effectively neutralizes immune-escape variants, while low-affinity sIgA impairs such neutralizing activity, and second, the integrity of sIgA’s secretory component (SC/SP)—stable binding between SP and the IgA heavy chain sustains long-term mucosal protection, whereas impaired SP integrity shortens the half-life of nasal mucosal sIgA and significantly reduces virus clearance efficiency [112]. This result clearly indicates that a single sIgA titer indicator cannot fully reflect mucosal protective efficacy; a comprehensive assessment incorporating sIgA antigen affinity and secretory component integrity is necessary to more accurately predict the clinical protective effect of intranasal vaccines.

In addition, pre-existing immunity refers to the innate or adaptive immune state established by the body through prior infection, vaccination, cross-reactivity and other means before receiving a new vaccination, pathogen infection or immunological intervention. It is a key confounding factor that must be prioritized in the research and clinical evaluation of mucosal vaccines and universal vaccines. A Phase II randomized clinical trial of the Russian-backbone intranasal live attenuated influenza vaccine (LAIV) conducted in Bangladesh revealed a significant negative correlation between pre-existing immunity levels and the strength of LAIV-induced humoral immune responses. The pre-existing immune status of vaccine recipients directly affected the viral shedding of the vaccine strain in the body: recipients with low pre-existing immunity exhibited significantly higher vaccine virus shedding rates, accompanied by more robust LAIV-induced humoral immune responses; in contrast, vaccine virus shedding was markedly suppressed in those with high pre-existing immunity, and the intensity of their humoral immune responses was correspondingly reduced [113]. Similar findings have been obtained in subsequent studies, which focused on the immunogenicity of the intranasal live attenuated influenza vaccine (LAIV) and clarified the critical impact of pre-existing immunity (including pre-existing antibodies) induced by the intramuscularly administered inactivated influenza vaccine (IIV) on the LAIV: while pre-existing antibodies do not impair LAIV-induced strain-specific immunity (e.g., protective effects against the homologous H1N1 CA04 virus), they significantly attenuate LAIV-mediated cross-protective efficacy against the heterologous H1N1 PR8 virus. Mice primed with the IIV followed by the LAIV exhibited markedly reduced protective efficacy upon a heterologous viral challenge compared to those vaccinated with the LAIV alone [114].

Currently, assessing pre-existing immunity related to intranasal vaccines and determining its impact follow a cohesive approach of baseline detection, stratified analysis and correlation verification: Nasopharyngeal swabs/nasal lavage fluid and peripheral blood serum are first collected to measure mucosal vaccine target-specific sIgA (including titer and antigen affinity), systemic specific IgG neutralizing antibody titers and memory immune cell subsets, thus establishing subjects’ pre-existing immune baselines. Subjects are then stratified by positive/negative and high/low pre-existing immunity, with post-vaccination mucosal and systemic immune response indicators (e.g., antibody fold increase, virus clearance efficiency, and vaccine virus shedding rate) measured and immunogenicity as well as protective efficacy compared across strata. Finally, statistical analysis verifies the correlation between baseline pre-existing immunity indicators and vaccine efficacy: pre-existing immunity is deemed to have a substantial impact on the intranasal vaccine if the high pre-existing immunity group shows significantly reduced vaccine-induced responses, inhibited vaccine virus replication, and markedly lower protective efficacy versus the non/low pre-existing immunity group, and vice versa. This indicator is therefore likely to play an important role in the future evaluation of intranasal vaccines [115].

From a regulatory perspective, as mucosal delivery systems, intranasal vaccines face more rigorous regulatory scrutiny than injectable formulations, with the lack of unified standards for mucosal safety evaluation being a major obstacle. For example, significant discrepancies exist in regulatory requirements for nasal mucosal irritation assessment: the U.S. Food and Drug Administration (FDA) mandates 12-week long-term mucosal histopathological studies in non-human primates, while the European Medicines Agency (EMA) only accepts 4-week data from rodents. This inconsistency prolongs approval cycles—with an average of 3–5 years for intranasal vaccines versus 1–2 years for injectable vaccines. Meanwhile, evaluating the safety of adjuvants poses substantial challenges. TLR agonists such as Poly I:C may induce excessive cytokine release, prompting regulatory authorities to often require additional pharmacodynamic studies to rule out systemic inflammatory risks and further increasing approval difficulties. Some small-molecule adjuvants exhibit dose-dependent toxicity: for instance, high-dose CpG ODNs (≥50 μg/dose) can cause necrosis of nasal mucosal epithelial cells in mice, with a mucosal damage rate as high as 20%. Although reducing the dose mitigates toxicity, it often compromises immunogenicity—lowering the CpG dose to 10 μg/dose results in a 40% decrease in the titer of sIgA in nasal lavage fluid [32]. Additionally, the long-term safety of biodegradable carriers remains unclear. Residual monomers of PLGA may accumulate in the nasal mucosa, triggering chronic low-grade inflammation, and 5-year long-term follow-up data in humans are currently lacking [67].

## 7. Future Directions and Promising Strategies

Building on the advances in adjuvants, carriers, and mucosal barrier mechanisms summarized above, addressing the unmet clinical needs and overcoming translational bottlenecks of intranasal vaccines require targeted innovative strategies. These future directions focus on integrating multi-technology advantages, adapting to clinical application scenarios, and resolving core contradictions between efficacy, safety, and accessibility—ultimately promoting the transformation of intranasal vaccines from laboratory research to routine clinical prevention tools.

Viral biomimetic carriers have emerged as a frontier in intranasal/vaccine adjuvants, by replicating viruses’ mucus-penetrating and immune cell-targeting abilities while reducing mucosal irritation and clearance risks associated with traditional carriers—thus effectively addressing the key challenges of poor mucus penetration and low immunogenicity and driving the shift from passive delivery to active adaptation to the nasal immune microenvironment. A representative innovation is the PLGA-PEG-Man nanocarrier owing to its mimicry of two key viral traits. The PLGA-PEG-Man nanocarrier achieves efficient nasal mucosal interaction: its polyethylene glycol (PEG) segment forms a hydrophilic “stealth” layer (replicating the glycosylated outer layer of viruses) to reduce mucus adhesion and enhance mucus penetration; the conjugated mannose ligands specifically bind to mannose receptors on the surface of antigen-presenting cells (mimicking viruses’ targeted binding to host cells), enabling efficient internalization and improved antigen presentation—this is the core of the viral biomimetic design. Constructed by the precise integration of biodegradable PLGA-PEG polymers with mannose ligands and endowed with dual functions of mucus penetration and active targeting, surface-conjugated mannose specifically binds to mannose receptors (MRs) on APCs to mediate endocytosis, while its pathogen-mimetic size enhances APC recognition and internalization. Notably, this design avoids excessive inflammation induced by conventional chemical adjuvants that directly activate TLR pathways, and its modularity allows for the flexible modification of targeting ligands as well as compatibility with protein subunit, inactivated virus, and nucleic acid vaccines—aligning with the demands for rapid iteration and multi-pathogen coverage in vaccine development [116].

Thermoresponsive intranasal vaccines, which are based on thermosensitive hydrogels (e.g., Poloxamer 407 and PNIPAM) and thermoresponsive nanocarriers, stand at the forefront of intelligent mucosal delivery, offering significant advantages in prolonged mucosal retention, antigen/nucleic acid stability, and targeting capabilities. Their modular design enables integration with mucus-penetrating and active targeting functions, allowing them to adapt to diverse APC subsets and mucosal sites while maintaining compatibility with multiple vaccine platforms [117].

pH-responsive intranasal vaccines are promising for targeted delivery and controlled release, with core advantages including antigen protection in the neutral nasal environment, reduced pulmonary deposition, and efficient antigen release in acidic endosomes. Both polymeric materials (e.g., poly(β-amino esters)) and inorganic nanocarriers (e.g., surface-modified mesoporous silica nanoparticles) can achieve pH responsiveness—protonation of amino groups in weakly acidic environments alters polymer hydrophilicity to trigger drug release [118]. Knight et al. synthesized pH-responsive diblock copolymers via RAFT polymerization; the nanoparticles conjugated with ovalbumin (OVA) promote endosomal escape upon acidification, enhance MHC-I pathway antigen presentation, and induce stronger antigen-specific CD8^+^ T-cell responses and lung tissue-resident memory T cell (TRM) expression compared to non-responsive carriers [119]. A major innovation is the integration of pH-responsive delivery with the neonatal Fc receptor (FcRn): fusing viral antigens with albumin enables FcRn-mediated transmembrane transport from the nasal mucosa to mucosa-associated lymphoid tissue (MALT), avoiding enzymatic degradation and pulmonary deposition. This formulation supports multi-antigen loading, induces long-term immune memory (100% survival of immunized mice against influenza for 4.5 months), and exhibits excellent biocompatibility with intranasal spray adaptability [118].

To address the core issues of insufficient vaccine cold chains and high wastage rates in low- and middle-income countries, the development of intranasal vaccines with high-temperature stability that can achieve efficient distribution independent of cold chains is also at the forefront of research. An innovative composite excipient system of “trehalose + leucine or trileucine” is adopted: trehalose protects the biological activity of antigens and lipid adjuvants through vitrification, while trileucine optimizes the surface morphology of particles (increasing rugosity). This not only enhances aerosol dispersibility but also assists in stabilizing protein structures, breaking through the limitation that a single excipient “cannot simultaneously balance stability and delivery efficiency [120,121]”. The intranasal vaccine, which was prepared as a dry powder via spray drying, has been proven to effectively solve this problem. The vaccine can be stably stored at 40 °C for 10 months and can withstand short-distance transportation without a cold chain, effectively reducing the loss of potency during storage. It is suitable for large-scale stockpiling and cross-regional distribution, which are especially important to adapt to the logistics scenarios of low- and middle-income countries. Additionally, this spray drying–vitrification stabilization system has been verified to be compatible with recombinant protein antigens and nanoliposomal adjuvants. In the future, by replacing antigens (such as influenza HA or anthrax PA), we can rapidly develop cold chain-independent intranasal vaccines against other respiratory pathogens [120].

A key recent trend in intranasal vaccination is unadjuvanted mucosal protein boosting, which harnesses pre-existing immunity (primary vaccination/natural infection) to eliminate exogenous adjuvants, a paradigm shift for safe and efficient mucosal immunization. Here, memory CD4^+^ T cells act as intrinsic “endogenous adjuvants”: nasal antigen re-exposure rapidly activates them in mucosal lymphoid tissues to secrete pro-inflammatory cytokines (e.g., IFN-γ, IL-2) and chemokines, recruiting and activating APCs (e.g., dendritic cells) to enhance antigen presentation. Direct CD4^+^ T-B-cell interactions (via CD40-CD40L and cytokines) also drive naive B-cell differentiation into sIgA-secreting plasma cells and prime CD8^+^ T cells into nasal/lung TRMs, the key mediators of rapid local antiviral immunity.

Kwon et al.’s recent study showed that intranasal delivery of unadjuvanted SARS-CoV-2 spike proteins after intramuscular mRNA-LNP priming elicits robust respiratory mucosal immunity via pre-existing immunity. Memory CD4^+^ T cells act as key endogenous adjuvants; their activation secretes CXCL9/CXCL10, recruiting lymph node-resident memory B cells to the lungs through CXCR3 signaling. These B cells differentiate into sIgA-secreting plasma cells via the CD40-TGFβ pathway, and memory CD4^+^ T cells also drive CD8^+^ T-cell conversion into TRMs. This adjuvant-free strategy induces durable mucosal immunity in the respiratory tract; repeated intranasal boosting amplifies the effect, with no endotoxin contamination, ensuring safety and efficacy. The study provides direct experimental evidence for next-generation intranasal vaccines, aligning with the field’s adjuvant-free and mucosal-targeting trends [122]. Mao et al. used the “prime and spike” (P&S) strategy and showed that intranasal administration of the unadjuvanted SARS-CoV-2 or SARS-CoV-1 spike protein after intramuscular mRNA-LNP priming induces strong protective respiratory mucosal immunity by exploiting pre-existing immunity. This approach elicits mucosal IgA and IgG responses and generates antigen-specific tissue-resident memory CD4^+^ and CD8^+^ T cells, as well as memory B cells, markedly reducing viral load and transmission with protection lasting at least 118 days. Being adjuvant- and viral vector-free, it improves safety; heterologous boosting also induces cross-protection against sarbecoviruses, reflecting the adjuvant-free and mucosal-targeted trends of intranasal vaccines [123]. Moriyama et al. demonstrated that the advanced strategy of intranasal unadjuvanted protein boosting under pre-existing immunity is also effective against influenza. Following intramuscular mRNA-LNP priming, intranasal administration of the unadjuvanted influenza HA protein induced strong respiratory mucosal immunity, eliciting mucosal IgA/IgG responses and tissue-resident memory T cells, which markedly reduced viral loads in both the upper and lower respiratory tracts. The approach was effective in aged mice and against heterologous strains. Without exogenous adjuvants, it provides key support for developing safe, mucosal-targeted, adjuvant-free intranasal influenza vaccines [124].

Combination adjuvants based on TLR agonists have also advanced intranasal vaccine development. Co-immunization with Poly I:C/CpG ODN adjuvants and H. pylori recombinant proteins (LpoB/UreA) significantly increases serum antigen-specific IgG (especially in Poly I:C and combined groups) and gastric sIgA levels, induces Th1-dominant immune responses, and enhances lymphocyte proliferation [125]. For SARS-CoV-2, the intranasal booster vaccine CP15–IN (combining CpG and Poly I:C) not only induces mucosal antibodies but also enhances trained innate immunity for faster pathogen clearance, with no obvious adverse reactions compared to single Poly I:C administration [44]. Additionally, Hiltonol^®^ (Poly I:C:LC)—a Poly I:C derivative stabilized with poly-L-lysine and carboxymethyl cellulose—retains TLR3/RIG-I/MDA5 activation, inhibits viral titers, induces neutralizing antibodies, and reduces adverse events in a mouse SARS-CoV model, with enhanced in vivo stability and prolonged action compared to unmodified Poly I:C [45].

Overall, the future breakthrough directions for intranasal vaccines should not be limited to the iteration of a single technology, but rather focus on the integration of multiple technologies and adaptation to clinical scenarios. The combination of smart responsive carriers (pH/temperature-responsive) and biomimetic design is expected to achieve the superimposition of multiple advantageous functions, including targeted delivery, efficient activation, and long-lasting immunity—this direction is worthy of focused investment. However, we must guard against excessive technical complexity: as the core value of vaccines lies in accessibility, overpursuit of cutting-edge technologies may lead to a surge in costs, decoupling from the needs of public prevention and control. In addition, the development of cold-chain-free formulations and multivalent broad-spectrum vaccines directly addresses the core pain points of global public health. Specifically, against the backdrop of frequent mutations in respiratory viruses, such “practical technological breakthroughs” hold greater public health value than merely improving immune indicators and should become key areas for the tilt of scientific research resources.

In addition to the aforementioned adjuvants already applied in intranasal vaccines, magnetic nanoparticles (magnetic particles with a size range of 1–100 nm and composed of magnetic materials such as iron, cobalt, nickel, and their oxides) offer an innovative approach for the targeted delivery of intranasal vaccines in future directions, with distinct core advantages: first, exceptional targeting ability (under the guidance of an external magnetic field, they can precisely accumulate at immune sites of the nasal mucosa, significantly improving the uptake efficiency of vaccines by immune cells and thereby enhancing local and systemic immune responses); second, superior safety (targeted delivery reduces the distribution of vaccines in non-target tissues, lowering the risk of systemic adverse reactions); and third, validated potential in biological applications (they can serve as carriers to achieve stable delivery of active ingredients).

Although there are currently no studies on magnetic nanoparticles as adjuvants for intranasal vaccines, relevant research on intranasal administration has provided experimental support for their application. A study by Seino et al. successfully achieved targeted delivery of magnetic nanoparticles to specific brain regions via intranasal administration, with approximately 0.3–0.6% of PEGylated magnetic nanoparticles migrating to the brain while retaining their magnetism, thus confirming the feasibility of intranasal delivery [126]. Notably, the intranasal administration route may pose potential risks: magnetic nanoparticles may cross the blood–brain barrier and enter the central nervous system (CNS) through the olfactory nerve pathway. If the particle size is excessively small (e.g., <100 nm) or the surface modification is inappropriate, they may accumulate in brain tissues, triggering neuroinflammation, cytotoxicity, or interfering with the normal physiological functions of the CNS. Therefore, future research should optimize the particle size, surface modification (e.g., adjusting the length of PEG chains), and administration dosage of magnetic nanoparticles. While retaining their advantages in targeted delivery, a tailored safety evaluation system for the CNS should be established to ensure the safety and efficacy of their application in intranasal vaccines.

## 8. Conclusions and Future Outlook

Significant breakthroughs have been made in the research on adjuvants and carriers for intranasal vaccines, laying a solid core foundation for the development of novel vaccines. In the field of adjuvants, TLR agonists have become the research focus due to their precise immune activation advantages, and the emergence of multi-adjuvant synergistic strategies has effectively resolved the balance dilemma between efficacy and safety faced by single adjuvants. In terms of carrier technologies, diverse approaches such as synthetic polymers, natural polysaccharides, and smart responsive materials continue to overcome core bottlenecks including mucosal penetration and stable antigen delivery. Essentially, the core challenge of current technologies is mainly reflected in the triangular balance between “immune efficacy, clinical safety and translational implementation”. The excellent performance achieved at the laboratory level must overcome practical hurdles such as large-scale production, quality consistency control, and regulatory standard adaptation to truly realize clinical translational value. Future research should move beyond the traditional mindset of “single-component optimization” and shift towards the in-depth integration of systematic synergistic innovation and clinical demand orientation. On the one hand, we should promote the integrated design of adjuvants and carriers to achieve a functional closed-loop of “targeted delivery; precise activation; long-lasting immunity”. On the other hand, we should also focus on practical clinical needs and strengthen breakthroughs in practical technologies such as cold-chain-free formulations and multivalent broad-spectrum vaccines. Only in this way can we accelerate the transformation of intranasal vaccines from emergency response tools to a routine prevention system for respiratory infectious diseases, thus providing a more efficient, convenient, and accessible technical solution for global public health prevention and control.

## Figures and Tables

**Table 1:** Summary of TLR agonists for intranasal vaccines: components, antigens, and immunological efficacy. Where ↑ and ↓ indicate an increase or decrease in the corresponding immune index levels, respectively.

Types of TLRs	Agonist Component	Antigens	Immune Efficacy/Immunological Effect	References
TLR9	CpG	EV71	Systemic/mucosal EV71 Abs ↑; serum/nasal/BALF/fecal IgG/IgA ↑; IgG2a ↑ (Th1-biased)	[32]
		SARS-CoV-2 Trimeric Spike Protein	Intranasal = subcutaneous protection; lung virus clearance ↑; neutralizing Abs/sIgA ↑; anti-variants, non-toxic	[33]
		M7	Serum IgG/IgM ↑; serum/nasal/fecal IgG1/IgG2c/IgA ↑; splenocyte IL-2/IFN-γ ↑; dLN memory T cells ↑; 100% survival	[34]
		SARS-CoV-2 Spike Protein (S Protein) Receptor-Binding Domain (RBD)	Th1-biased mucosal immunity; neutralizing Abs (serum/BAL) > alum	[35]
TLR5	Salmonella Flagellin	EXP153-rFliC Conjugate Peptide	Th1-enhanced (Th2-dominant); systemic/mucosal EXP153 Abs ↑	[36]
	FliC	CT, Uropathogenic Escherichia coli (UPEC)-Expressed Protein FimH	FimH Th1/Th2 ↑; intranasal immunity > subcutaneous; FimH.FliC: ↑ immunogenicity/UPEC clearance; long-lasting IgG ↑	[37]
TLR4	PS-G	EV-A71	Respiratory/mucosal IgA ↑; systemic/mucosal cross-protection ↑; broad EV-A71 coverage; non-toxic	[38]
	Polymeric Inulin Acetate Nanoparticles (InAc-NPs)	OVA	Potent systemic and mucosal immunity induced by InAc-NPs. Markedly enhanced antigen-specific IgG1/IgG2a versus PLGA control. Robust mucosal sIgA across respiratory and intestinal sites. Strong immune memory with humoral/cellular activation	[39]
	PHAD (Phosphorylated HexaAcyl Disaccharide)/3D-PHAD^®^	Respiratory Syncytial Virus (RSV)	RSV-specific neutralizing Abs and IgG ↑; high adjuvant activity at low dosage (50% adjuvant reduction); excellent formulation compatibility and long-term stability (300 days at 4 °C)	[40]
	GLA (Glucopyranosyl Lipid A)	*Mycobacterium tuberculosis* (Mtb) Recombinant Antigen ID93	Systemic ID93-specific IgG ↑; pulmonary mucosal IgA and polyfunctional Th1-type CD4^+^ T-cell responses ↑; reduced pulmonary bacterial load and granuloma area; protective efficacy > BCG	[41]
	BECC (Bacterial Enzymatic Combinatorial Chemistry) (BECC438, BECC470)	Amebiasis Antigen	Robust and durable immune responses; efficient targeted deposition in adult/infant nasal cavity; low lung penetration; synergistic effect with 3M-052	[42]
		Recombinant Hemagglutinin (rHA)	Balanced Th1/Th2 immune response (IgG1/IgG2a ↑); superior protective efficacy against homologous (NL/09 H1N1) and heterologous (Sing/2015 H1N1) influenza A virus; antigen/adjuvant-sparing effects (7-fold antigen reduction and 10-fold adjuvant reduction)	[43]
	Poly I:C;CpG	Spike (S) Protein of SARS-CoV-2 Original Strain Wuhan-1 (WA-1) and Beta (B.1.351) Variant	Alum prime + nasal boost; mucosal Ab retained; trained innate immunity ↑; nasal virus cleared; non-toxic (dual adjuvants)	[44]
	Poly I:C:LC	SARS-CoV	Viral titer ↓; neutralizing Abs ↑; mouse adverse reactions ↓; stable, slow degradation, and long-acting	[45]

## Data Availability

No new data were created or analyzed in this study. Data sharing is not applicable to this article.
